# A Hairy Cituation – PADIs in Regeneration and Alopecia

**DOI:** 10.3389/fcell.2021.789676

**Published:** 2021-12-13

**Authors:** Kim Vikhe Patil, Kylie Hin-Man Mak, Maria Genander

**Affiliations:** Department of Cell and Molecular Biology, Karolinska Institutet, Stockholm, Sweden

**Keywords:** citrullination, hair follicle, alopecia, inflammation, stem cell, epigenetics, Peptidylarginine deiminase

## Abstract

In this Review article, we focus on delineating the expression and function of Peptidyl Arginine Delminases (PADIs) in the hair follicle stem cell lineage and in inflammatory alopecia. We outline our current understanding of cellular processes influenced by protein citrullination, the PADI mediated posttranslational enzymatic conversion of arginine to citrulline, by exploring citrullinomes from normal and inflamed tissues. Drawing from other stem cell lineages, we detail the potential function of PADIs and specific citrullinated protein residues in hair follicle stem cell activation, lineage specification and differentiation. We highlight PADI3 as a mediator of hair shaft differentiation and display why mutations in *PADI3* are linked to human alopecia. Furthermore, we propose mechanisms of PADI4 dependent fine-tuning of the hair follicle lineage progression. Finally, we discuss citrullination in the context of inflammatory alopecia. We present how infiltrating neutrophils establish a citrullination-driven self-perpetuating proinflammatory circuitry resulting in T-cell recruitment and activation contributing to hair follicle degeneration. In summary, we aim to provide a comprehensive perspective on how citrullination modulates hair follicle regeneration and contributes to inflammatory alopecia.

## Introduction

Hair follicles (HFs) undergo phases of destruction and regeneration throughout the lifespan of an organism. Regeneration and hair formation depend on balanced stem cell renewal and differentiation, integrating transcriptional and epigenetic regulation with microenvironmental niche-derived cues. Failure to coordinate signaling, or respond to inflammatory signals, deregulates hair follicle lineage progression, abrogates regeneration, and commonly leads to alopecia. Posttranslational modifications are enzymatically catalyzed amino acid alterations, which constitute a non-genetic mechanism for modulating protein function. Whereas our understanding of how posttranslational modifications affects stem cell lineage progression and disease progression is far from complete, extensive work identifying histone modification “codes” acting to maintain cell states as well as to ensure proper lineage progression, suggest that the impact of protein modifications requires further investigation.

Here, we focus on how citrullination contributes to hair follicle lineage progression during regeneration, and how citrullination-mediated inflammation acts to perpetuate disease progression in inflammatory alopecia. We hope that this work will inspire scientists to explore new citrullination paved avenues towards the understanding of skin and skin disease.

### Peptidylarginine Deiminases Catalyse Citrullination

**FIGURE 1:**
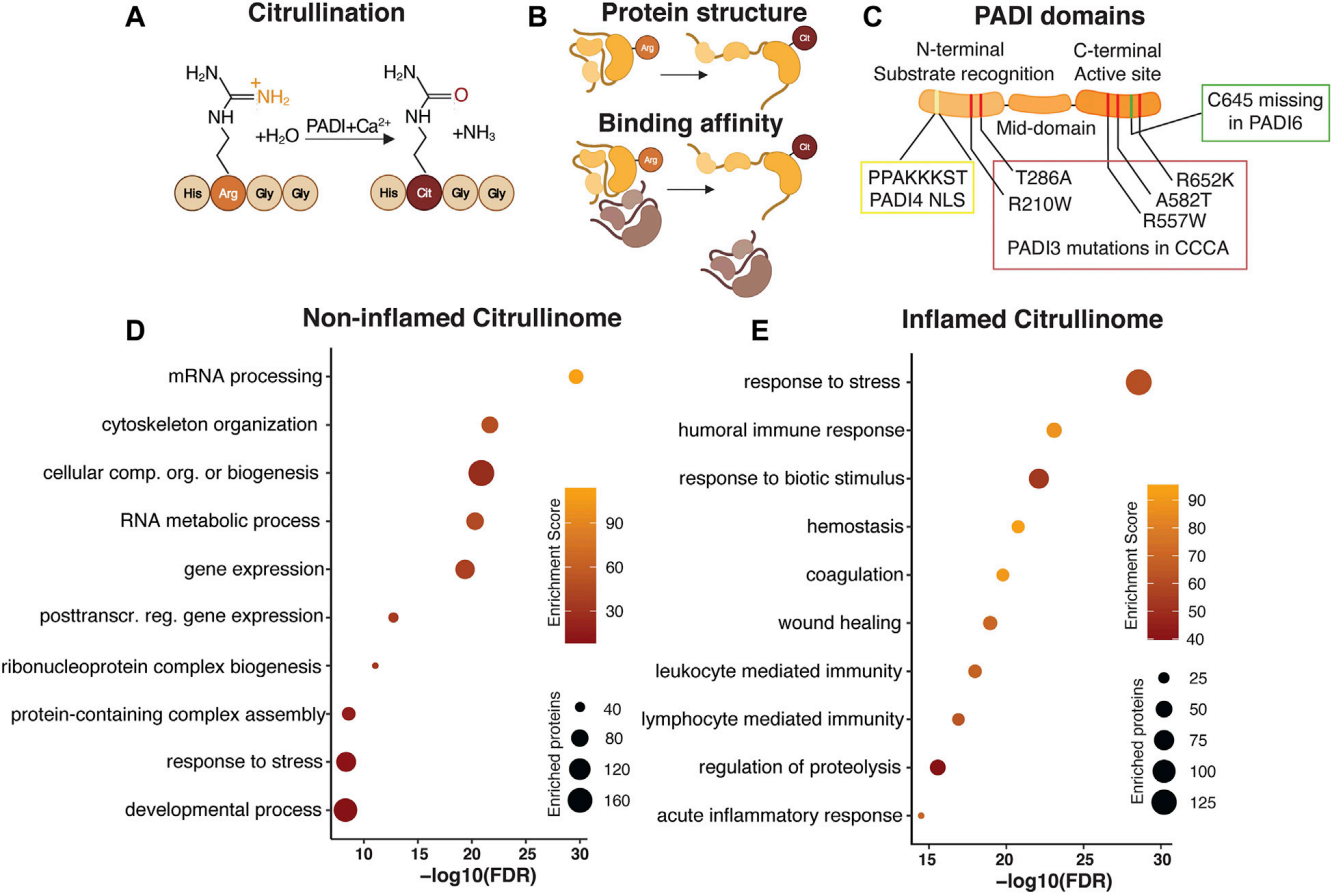
Citrullination as a dynamically regulated posttranslational modification. Citrullination, the Peptidylarginine deiminase (PADI) dependent conversion of arginine to citrulline **(A)** is an irreversible posttranslational modification. PADIs require the presence of calcium for the enzymatic activity, which acts to replace the amide group of the arginine side chain with oxygen, yielding ammonia as a side product. Replacing the positively charged arginine with neutral citrulline alters the protein conformation and binding affinity **(B)**. PADI enzymes are highly conserved with an N-terminal domain mediating substrate recognition, a middle domain, and a C-terminal domain where the active site resides. The N-terminal domain in PADI4 contains a nuclear localization signal (NLS). Human *PADI3* mutations associated with CCCA are mapped to both the N- and C-terminal domains. Cysteine 645, essential for active site catalytic activity is missing in PADI6 **(C)**. Gene Ontology analysis of published human citrullinomes display distinct enrichment profiles in non-inflamed compared to inflamed tissue **(D,E)**.

Protein citrullination, or deimination, is the conversion of the positively charged amino acid arginine to neutral citrulline by replacement of the arginine side chain imine with an ureido group (Figure 1A). Peptidylcitrulline, the presence of the non-essential amino acid citrulline, is thus not a product of translation but generated *via* enzymatic alteration of an existing peptide.

Catalysing citrullination are the highly conserved, calcium dependent, Peptidylarginine deiminase enzymes. There are currently five different mammalian PADIs described, namely PADI1, 2, 3, 4, and 6. PADIs generally require calcium binding to bring about conformational changes that generate the active cleft and enzymatic activity. Once active, PADI enzyme activity alters the overall charge, conformation and function of the target protein (Figure 1B) (Vossenaar et al., 2003). Although PADIs display up to 95% of sequence homology (Mechin et al., 2007) they show distinct tissue expression, localization and dimerization ability (Vossenaar et al., 2003), all which collectively affects protein substrate specificity. Whereas PADI1, 3 and 6 localizes to the cytoplasm, PADI2 and PADI4 can shuttle to the nucleus. In addition, PADI2/3/4, but not PADI1, attain full enzymatic activity only after a head-to-tail (N-to-C domain) homodimerization (Arita et al., 2004; Lee C.-Y. et al., 2017; Saijo et al., 2016; Slade et al., 2015). Interestingly, PADI6 lacks several calcium binding sites as well as the active cleft amino acid cysteine 645, which are conserved in the other family members (Witalison et al., 2015) and is unable to citrullinate substrates which are readily citrullinated by the other PADIs *in vitro* (Knuckley et al., 2010) suggesting that the lack of C645 renders PADI6 enzymatically inactive (Figure 1C). It is however possible that PADI6 has different substrate preferences or requires additional cofactors than calcium to function (Raijmakers et al., 2007). PADI6 homodimerization is yet to be confirmed.

The catalysation of citrullination commonly either antagonizes or facilitates other types of posttranslational modifications, such as methylation and acetylation (Cuthbert et al., 2004; Denis et al., 2009), thereby extending the functional impact of citrullination well beyond single arginine residue modification. Interestingly, whereas other posttranslational modifications are reversible, no de-citrullinating enzyme is yet described, suggesting that removal of citrullination is linked to protein turnover.

### Mining the Citrullinome to Understand Function

Most work detailing citrullination have reported the identification of single citrullination residues, and successfully elucidated the functional impact of the isolated modification on that particular protein in a specific context. In more recent attempts to identify cellular processes influenced by the enzymatic action of PADIs, the citrullinome, or proteome-wide citrullination signature, of distinct cell types, organs and disease conditions have been characterized by mass spectrometry. Focusing on human citrullinomes, we analysed all citrullinated proteins identified in normal non-inflamed cells and tissues (Lewallen et al., 2015; Lee et al., 2018; Tanikawa et al., 2018) and compared their functional classification to citrullinated targets found in inflammatory disease (370 and 251 unique proteins respectively) (van Beers et al., 2013; Tutturen et al., 2014; Tilvawala et al., 2018). Selecting for the 10 most enriched, and unique, gene ontology terms reveal striking differences in biological processes associated with, and hence likely to be influenced by, citrullination. Whereas citrullination in non-inflamed tissue is associated with, but not limited to, mRNA processing, gene expression and cytoskeletal organization, protein citrullination in inflammatory disease is centred around stress and immune responses (Figures 1D,E). It is interesting that the biological processes linked to citrullination are distinct in normal and inflamed tissue, hinting at key cellular functions where citrullination is required for maintaining cell identity in non-inflamed tissue, as well as identifying aspects of citrullination-dependent biology which could be explored for therapeutic purposes during inflammation. Collectively, this meta-analysis indicates that the citrullinome is not a static entity, but rather dynamically defined, relying on context and cell type specific determinants to distinguish the biological processes influenced by citrullination.

### How PADIs Find Their Targets – What we Know About Substrate Specificity

Despite the abundance of protein arginine, not all proteins, and far from all arginine residues become citrullinated. How PADI enzymes determine substrate specificity, and if substrate specificity is altered during disease, like inflammatory alopecia, is largely unknown.

PADI1/3 mediated citrullination in the HF is associated with remodeling of intermediate filaments, usually by affecting filament polymerization, susceptibility to cross-linking or proteolytic enzymes and dimerization abilities (Kizawa et al., 2008; Briot et al., 2020), functionality required for hair shaft differentiation. Interestingly, many PADI1/3 known substrates belong to the S100-fused protein family (filaggrins, hornerin, trichohyalin and S100A3), hinting at an overlapping preference based on function and/or structure for substrate specificity (Tarcsa et al., 1997; Knuckley et al., 2010). However, *in vitro* citrullination of S100A3 demonstrates that PADI1 and PADI3 citrullinate distinct S100A3 arginine residues (Kizawa et al., 2008), suggesting that PADI1/3 citrullination specificity is more complex than general affection for S100-fused proteins. If PADI enzyme substrate specificity is altered during the development of inflammatory alopecia is a matter of speculation, however pro-inflammatory cytokines have been demonstrated to inhibit epidermal PADI1 expression, subsequently reducing citrullination of KRT1 and contributing to remodeling of the inflamed epidermis (Padhi et al., 2021). In addition, proteomic analyses demonstrate epidermal specific citrullination profiles in atopic dermatitis (Winget et al., 2016), suggesting that alterations of both enzyme and substrate abundance are common features of skin inflammation.

Whereas arginine residues on all histones tails have been shown to be citrullinated (Cuthbert et al., 2004; Wang et al., 2004; Hagiwara et al., 2005), only H1 and H3 residues (H1R54 and H3R2/R8/R26) are jointly citrullinated by PADI2 and PADI4 (Christophorou et al., 2014; Falcao et al., 2019). Similarly, only a minority of PADI2 and PADI4 non-histone targets are shared (Tanikawa et al., 2018), suggesting that the function of PADI enzymes is largely non-redundant. Clues to enzyme specificity come from work identifying an RG/RGG motif in a subset of PADI4 targets (Tanikawa et al., 2018) indicating that binding motifs determining enzyme target specificity may exist.

**FIGURE 2:**
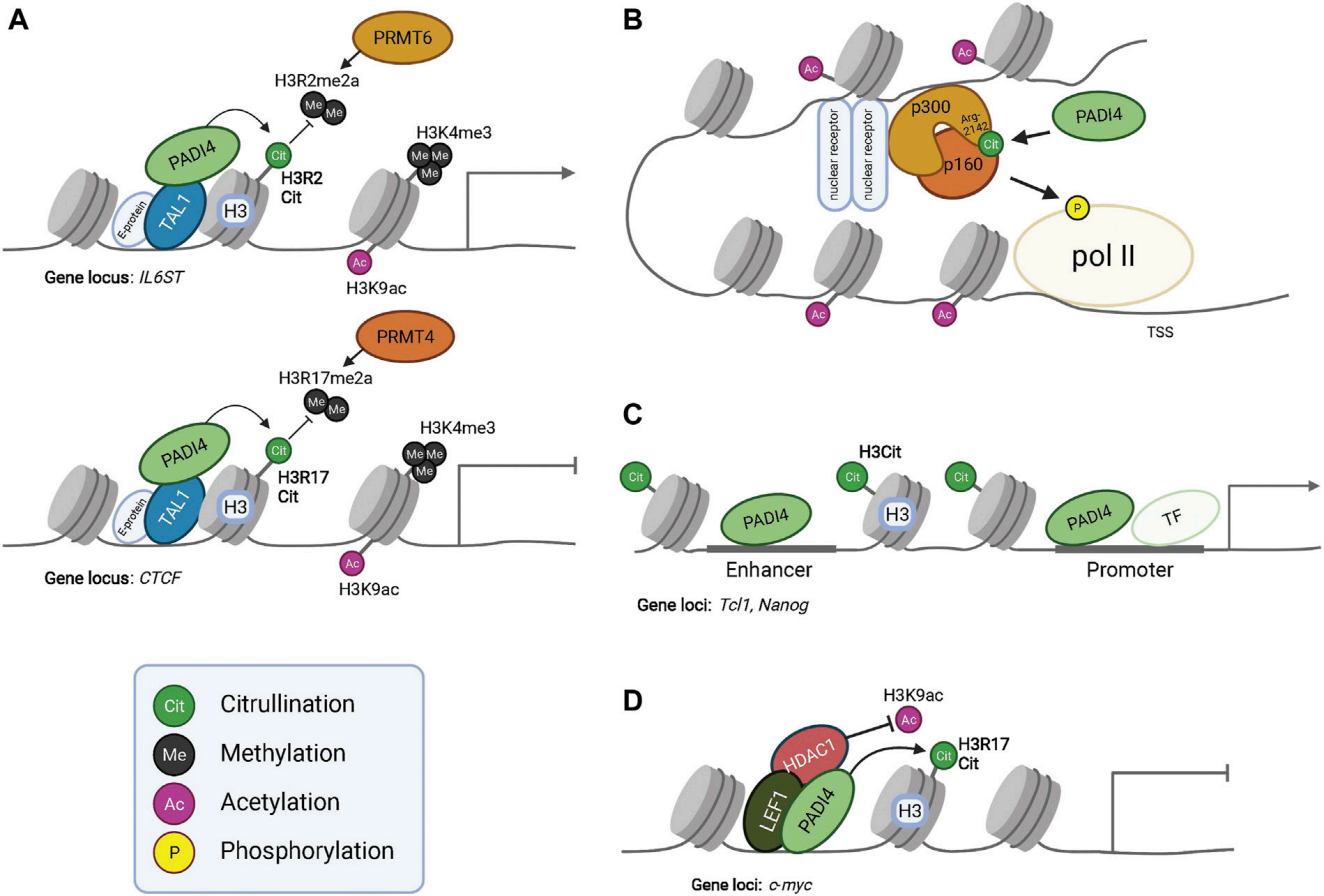
PADI dependent modulation of epigenetic and gene expression programs. PADI4 interacts with transcription factor TAL1 and citrullinates histone H3 to regulate gene expression. Citrullination of H3R2 results in antagonism of PRMT6 mediated methylation at this residue, resulting in the activation of IL6ST transcription **(Upper panel** in **A)**. Citrullination of H3R17 antagonizes PRMT4 mediated methylation and represses gene transcription of CTCF **(Lower panel** in **A)**. PADI4 can in this way function both as a repressor or activator of gene expression, given the context and binding partners. However, PADI4 always antagonizes the function of PRMTs. The active transcription mark H3K9ac is present in both conditions but at much lower levels at the CTCF locus, indicating interplay between histone tail modifications for transcriptional regulation. **(B)** Citrullination of acetyltransferase p300 at residue R2142 by PADI4 facilitates its dimerization with p160, leading to histone acetylation and eventually phosphorylation of Polymerase-II (pol II) for activated gene transcription. Additionally, citrullination of R2142 antagonizes its methylation by PRMT4 (CARM1) (not shown), exemplifying the regulatory effects of the subtle yet significant post-translational modifications. **(C)** Occupancy of PADI4 in combination with histone H3 citrullination at the enhancer and promoter of *Nanog* and *Tcl1* enable gene expression. **(D)** PADI4 can interact with HDAC1 and together bind to LEF1 at the *c-myc* transcriptional start site to repress gene expression. During PADI4-deficiency, HDAC1 fails to bind LEF1 and *c-myc* repression is alleviated.

Functionally, the outcome of histone citrullination is diverse. Citrullination of linker histone H1 (H1R54) result in chromatin de-condensation and transcriptional activation (Christophorou et al., 2014; Guertin et al., 2014), whereas H3 citrullination (H3R2, R8, R17 and R26) is associated with both transcriptional repression and activation, likely by counteracting activating methylation marks or by recruitment of additional histone-modifying enzymes (see Figure 2). Furthermore, PADI2/4 mediated citrullination of transcription factors (Kolodziej et al., 2014; Ghari et al., 2016; Sun et al., 2017; Sun et al., 2019) or epigenetically active enzymes (Lee et al., 2005; Christophorou et al., 2014; Deplus et al., 2014) allows PADIs to influence cell states independent of histone citrullination.

Skin immune cell infiltration is a hallmark of inflammatory alopecia. Activated neutrophils and T-cells express enzymatically active PADI2 and PADI4 which contributes to several aspects of the inflammatory process. For example, H3R8 citrullination at pro-inflammatory TNF-α and IL-8 promoters drive cytokine expression (Sharma et al., 2012) in activated T-cells. In addition, direct citrullination of IL-8 and TNF-α proteins modulates their pro-inflammatory effect (Proost et al., 2008; Moelants et al., 2013) highlighting the many ways cell state and type can influence citrullination specificity.

Even though we do not fully understand PADI enzyme substrate specificity, it is clear that individual enzymes display distinct substrate preferences. Although tissue and disease-associated alterations in the citrullinome correlate to enzyme activity and substrate abundance, aspects of substrate recognition may be context-dependent, relying on, for example, a tissue-specific set of co-factors.

### Citrullination and Stem Cell Renewal

Here we aim to delineate the function of PADIs from a hair follicle-centric perspective, using the wealth of existing hair follicle stem cell (HFSC) expression profiling as the starting point for understanding the impact of citrullination on HFSC lineage progression. A functional role for PADIs in epidermal differentiation is well established and comprehensively summarized elsewhere (Cau et al., 2018; Mechin et al., 2020).

As hair follicles (HFs) initiate regeneration, primed hair germ stem cells are the first to divide, followed by proliferation of bulge HFSCs (Greco et al., 2009). Whereas profiling of resting HFs reveals little evidence of *Padi* expression, mRNAs of both *Padi3* and *Padi4* are found in activated hair germ and bulge stem cells (Lien et al., 2011) and *Padi3/4* chromatin accessibility is increased in regenerating compared to resting hair germ (Adam et al., 2018), suggesting that PADI expression, and potentially function, is coupled to HFSC activation and self-renewal. Whilst there is no functional genetic evidence available for the role of *Padi3/4* in HFSCs, *Padi4* is part of the hematopoietic stem cell self-renewal gene signature (Nakashima et al., 2013; Young et al., 2021), and *Padi4* KO mice display increased hematopoietic stem cell proliferation (Young et al., 2021) indicating a PADI4-dependent program maintaining the stem cell pool by restricting proliferation and self-renewal. Work in embryonic stem cells reveal that PADI4 is required for maintaining pluripotency and loss, or chemical inhibition, of PADI4 leads to arrested embryo development, reduced expression of pluripotency genes and skewing of fate towards differentiation (Kan et al., 2012; Christophorou et al., 2014; Zhang et al., 2016). It is possible that the presence of PADI3/4 in activated HFSCs act complementary to restrict proliferation and maintain the stem cell pool during hair follicle regeneration.

Interestingly, PADI4 associates with, and citrullinates, *de novo* DNA methylases DNMT3A and DNMT3B (Christophorou et al., 2014; Deplus et al., 2014). DNMT3A and DNMT3B are found in HFSCs located in the bulge and in the outer root sheet during hair follicle regeneration (Rinaldi et al., 2017; Joost et al., 2020). Whereas HFSC expression of *Dnmt3a/b* increases during telogen-to-anagen transition (Lien et al., 2011), no significant *Dnmt3a/b* expression is found in progenitor or differentiated lineages of the regenerating HF, indicating that the function of DNMT3A/B is restricted to HFSCs. DNA methylation signatures of quiescent and activated HFSCs are distinct and sufficient to define the two stem cell states (Bock et al., 2012), suggesting that the DNMT3 activity is dynamically regulated during HFSC activation and subsequent initiation of HF growth. PADI4 mediated citrullination of arginine residues in the DNMT3A nuclear localization signal leads to increased DNMT stability, methyltransferase activity (Deplus et al., 2014; Sun et al., 2017; Zeng et al., 2020) and likely affects DNMT3A binding affinity to other proteins (Guo and Fast, 2011; Stadler et al., 2013), indicating that PADI4 could influence global DNA methylation patterns by targeting DNA methylases in activated HFSCs.

Loss of DNMT3A in quiescent HFSCs fails to induce HFSC activation (Chen et al., 2021). However, human epidermal stem cells devoid of either DNMT3A or DNMT3B display reduced self-renewal and increased differentiation upon grafting (Rinaldi et al., 2016). It is possible that *de novo* methylation is largely dispensable in quiescent HFSCs, however required when HFSCs are challenged to self-renew during HF regeneration. If so, PADI4-mediated citrullination in renewing HFSCs could act to modulate and/or physically direct DNMT3 methylase activity, ensuring balanced stem cell renewal and proliferation during initiation of hair follicle regeneration.

### Expression and Function of PADIs in Regenerating Hair Follicles

Activation and expansion of hair germ stem cells leads to the formation of the hair bulb, consisting of progenitor cells which specify into subpopulations of lineage committed progenitors, most of which eventually exit the cell cycle and fuel the differentiated hair lineages. Single cell RNA-sequencing place *Padi1*, *Padi3* and *Padi4* in discrete cell lineages during hair differentiation (Joost et al., 2020). Whereas *Padi1/3* expression is mapped to the differentiated inner root sheath (IRS) and medulla lineages, *Padi4* is preferentially expressed in the cortical hair shaft and medulla lineages (Adam et al., 2018; Joost et al., 2020). In addition, *Padi3* and *Padi4* are turned on in the hair bulb progenitor population, likely before lineage commitment (Nachat et al., 2005; Adam et al., 2018; Joost et al., 2020) and judging from the mutually exclusive expression pattern in IRS and cortical lineages, it is possible that PADI3 and PADI4 are involved in the specification of hair shaft progenitors.

Interestingly, *Padi3* and *Padi4* chromatin is bound by combinations of BMP and WNT lineage effectors in HF progenitor and committed lineage cells. Whereas PADI3 is induced in response to Vitamin D Receptor and LEF1 (independent of β-Catenin activation) (Palmer et al., 2008), LEF1 ChIP-sequencing in HF progenitor cells identifies *Padi4*, but not *Padi3*, as a LEF1 target gene (Adam et al., 2018). Furthermore, pSMAD1/5 binding sites are located to both *Padi3* and *Padi4* promoters (Genander et al., 2014), indicating that aspects of WNT signaling, in cooperation with BMP is required for fine-tuning of *Padi3* and *Padi4* expression in the regenerating hair follicle. Collectively, these data suggest that expression of PADI3/4 is under the control of specific subsets of hair follicle lineage effectors, potentially acting to further refine or lock lineage specification by mediating PADI expression.

**FIGURE 3:**
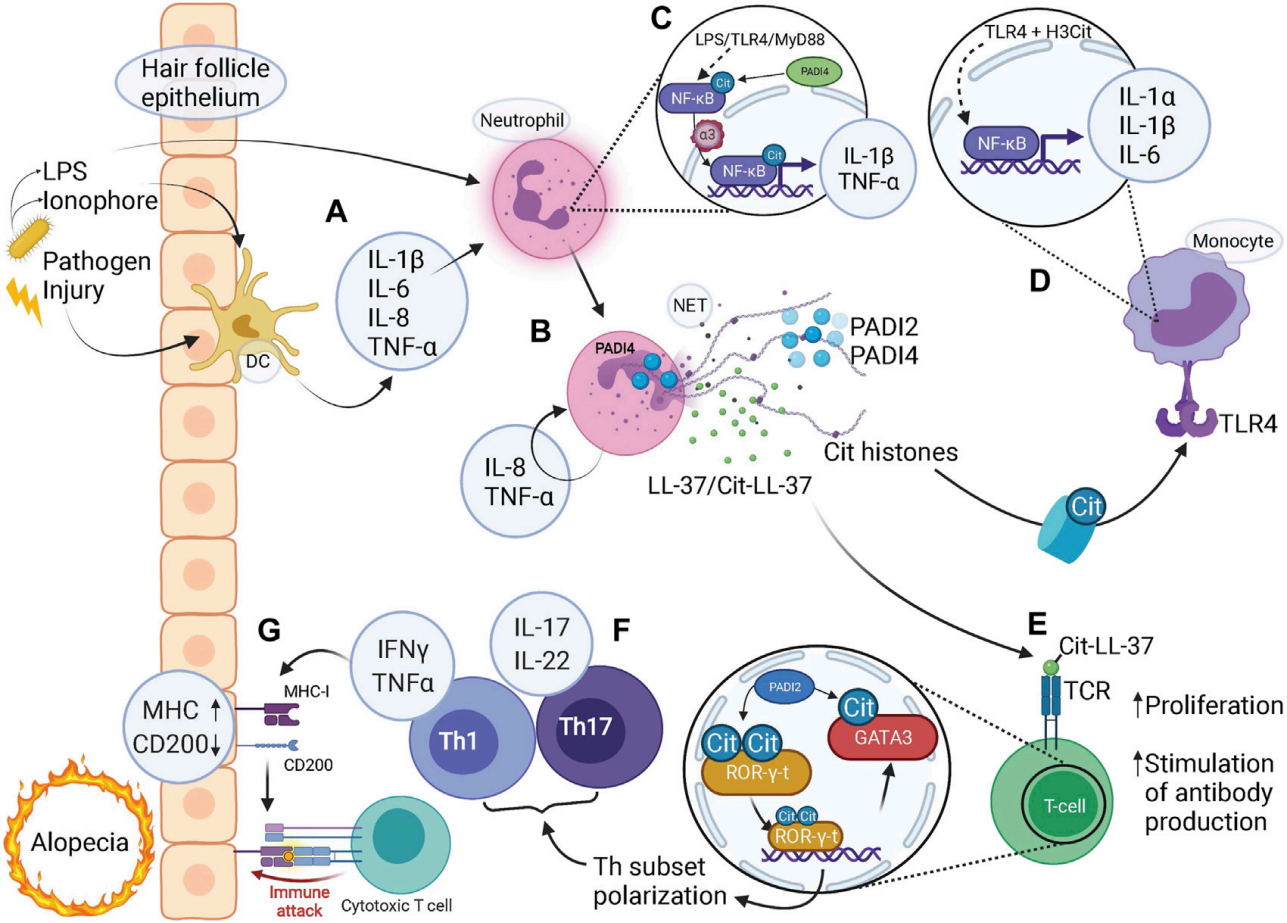
Citrullination-mediated inflammation contributes to alopecia. **(A)** Microbial or damaging insults stimulate tissue residing dendritic cells (DC) to release pro-inflammatory cytokines which in turn activates neutrophils. Pathogen-derived LPS and ionophores can also act to directly stimulate neutrophils *via* TLR4-binding or influx of calcium, respectively. **(B)** NETs released *via* PADI4-dependent pathways by activated neutrophils contain citrullinated (Cit) histones, PADI2, PADI4, LL-37 and citrullinated LL37 (along with granular components such as MPO and NE, not shown). **(C)** LPS binding to TLR4, acting *via* MyD88, activates NF-κB signaling. Citrullination of NF-κB by PADI4 enhances its interaction with importin-α3 which mediates nuclear translocation of NF-κB, with expression of pro-inflammatory target genes IL-1β and TNF-α. **(D)** Citrullinated histones bind to TLR4 and *via* NF-κB signaling elicit a strong inflammatory response by release of pro-inflammatory cytokines IL-1α, IL-1β, and IL-6. **(E)** Citrullinated (Cit)LL-37 generated during NET release is a strong TCR agonist, which enable increased T-cell proliferation and facilitates T-cell stimulated production of anti-cit/native cross-reactive LL-37 autoantibodies. **(F)** Citrullination of transcription factors ROR-γ-t and GATA3 in T-helper (Th) cells skews cell fate towards Th1 and Th17 subsets with associated pro-inflammatory cytokine profiles. **(G)** A pro-inflammatory milieu coerces the hair follicle epithelium to upregulate MHC class-I to present autoantigens as well as downregulate “no-danger” signal CD200, resulting in a collapse of immune privilege. Given the activated state of the adaptive immune system, represented by T-cells, an immune attack is imminent, bringing about either destruction or exhaustion of HFSCs resulting in alopecia. **(A–G)** Note the several steps at which pro-inflammatory cytokines are produced to facilitate an inflammatory environment, involving both the innate and adaptive immune system either on their own or in combination, highlighting the contribution of citrullination at all levels of immunity and inflammation.

Analogously, PADI2 skews effector T-cell specification towards a Th17 fate at the expense of Th2 (Kawalkowska et al., 2016; Sun et al., 2019), mechanistically by citrullination of arginine residue R330 located in the DNA binding domain of the transcription factor GATA3. Citrullinated GATA3 fails to bind DNA efficiently, resulting in reduced expression of GATA3 target genes and subsequent Th lineage fine-tuning (Sun et al., 2019) (Figure 3). Interestingly, GATA3 is required for IRS progenitor specification (Kaufman et al., 2003; Genander et al., 2014) indicating that co-expression of PADI4 and GATA3 in a subset of progenitor cells could act to inhibit the transcriptional activity of GATA3, hence favoring hair shaft over IRS lineage specification.

In addition to citrullination of transcription factors and consequently modulating their binding affinity towards DNA, other co-factors or their subcellular localization (Christophorou et al., 2014; Ghari et al., 2016; Sun et al., 2017; Zhang et al., 2011), PADI4 can, on its own, act as a transcriptional regulator. For example, PADI4 interacts with transcription factor TAL1 at gene promoters in a leukemic cell line (Figure 2A) (Kolodziej et al., 2014) acting to facilitate activation as well as silencing of lineage determining genes, likely by recruiting distinct co-factors at different subsets of genes.

### PADIs in Hair Follicle Lineage Differentiation

Although *Padi1/3* are co-expressed in the differentiated inner root sheath and medulla lineages, *Padi1* expression is significantly lower than that of *Padi3,* identifying PADI3 as the likely main contributor of protein citrullination in the IRS and medulla. PADI1/3 protein localization correlates well with mRNA expression, as well as global citrullination pattern in both human and mouse hair follicles (Nachat et al., 2005; Kizawa et al., 2008; Palmer et al., 2008), supporting the spatial resolution of the transcriptional profiling. It is not clear what regulates *Padi1* expression in the hair follicle, however *in vitro* data suggests Vitamin D signaling to be upstream of *Padi1* transcription in the epidermis (Hu et al., 2014; Padhi et al., 2021).

Functionally, PADI3 has been shown to citrullinate trichohyalin (TCHH) (Rogers et al., 1977; Tarcsa et al., 1996; Tarcsa et al., 1997), a structural protein abundantly expressed in the IRS and medulla. It is believed that PADI1/3-mediated citrullination of trichohyalin provide mechanical strength and enable air entrapment for thermal insulation in the IRS and medulla, respectively (Steinert et al., 2003). Citrullination allows trichohyalin solubilization from granules and subsequent transglutaminase-3 (TGM3) mediated crosslinking to keratins, further contributing to the hair shaft structure (Tarcsa et al., 1997). Other established citrullinated proteins expressed in the HF include the shared PADI1/3 structural protein target S100A3 (Kizawa et al., 2008) also associated with hair shaft differentiation.

Human *PADI3* mutations are linked to uncombable hair syndrome, manifesting as frizzy and fair hair resistant to combing flat (Basmanav et al., 2016), and Central centrifugal cicatricial alopecia (CCCA), a type of scarring alopecia found predominantly in women of African ancestry (Malki et al., 2019). *PADI3* mutations affect both protein folding and enzymatic activity, which manifests functionally (Figure 1C). Uncombable hair syndrome is caused by the lack of crosslinking of intermediate filaments in the IRS (Tarcsa et al., 1997) and CCCA patients display reduced levels of both trichohyalin and S100A3, suggesting that reduced PADI3-mediated citrullination affects both target function and stability in the human hair shaft.

In contrast to the enrichment of PADI3 found in the IRS, *Padi4* is preferentially expressed in the cortical hair shaft lineage together with the medulla (Adam et al., 2018; Joost et al., 2020). Whereas PADI4 is associated with self-renewal and stemness in some systems, nuclear PADI expression is also positively linked to differentiation. PADI2 expression drives oligodendrocyte differentiation, and expression is increased with differentiation (Falcao et al., 2019). Similarly, differentiated macrophages display higher PADI2/4 levels compared to the undifferentiated U937 monocyte cell line (Lai et al., 2019). Considering that PADI2/4 can have common targets (Tanikawa et al., 2018), it is possible that PADI2 and PADI4 have redundant functions in systems where co-expressed. In contrast, PADI4 is not reported to be able to compensate for PADI3, likely due to their mutually exclusive cellular localization, suggesting that PADI1/3 and PADI4 have unique targets and hence functions in hair follicle lineage differentiation.

### PADIs as Modulators of the Epigenetic Landscape

In addition to directly citrullinating DNMTs and affecting DNA methylation patterns, emerging evidence suggest that PADI4, and to some extent PADI2, can through various mechanisms impinge on the epigenetic landscape by modulating enhancer availability as well as histone modifications. Here we delineate a PADI4 mediated epigenetic interplay relevant for hair follicle regeneration.

To allow cell state specific combinations of key transcription factors to drive hair follicle lineage progression, HFSC fate transitions require dynamic remodeling of enhancers (Adam et al., 2015). Interestingly, Lee et al. demonstrated that the enhancer assembly of p300 acetyltransferase transcriptional coactivator complex is controlled by the counteracting effects of PADI4-mediated citrullination and PRMT4-mediated methylation, at arg-2142 residue of p300 (Lee et al., 2005). Methylation of arg-2142 by PRMT4 blocked co-factor p160 binding to p300, thereby inhibiting the acetylation activity of p160/p300 and transcription of target genes. In contrast, citrullination of p300 restored p160 binding *in vitro* and activated target gene transcription (Figure 2B) (Shang et al., 2000).

Although the HF might not rely on the steroid receptor p160 as a transcriptional coactivator, the above finding indicates citrullination of p300, a general acetyltransferase, controls both chromatin organization and transcriptional regulation by interfering with co-factor binding. It is possible that LEF1, in accordance with its role in keeping cell-type specific enhancers active during HFSC lineage commitment and lineage diversification (Adam et al., 2018), could act as the HF specific p300 co-activator.

Additionally, ChIP-qPCR in mouse embryonic stem cells revealed that PADI4 can target to enhancers. Occupancy of PADI4 was detected at the enhancer of *Tcl*, *Nanog*, and *Kit*, and H3 citrullination of these loci was sharply reduced upon treatment with the PADI4 inhibitor Cl-amidine, strongly suggesting that PADI4 is an enhancer-associated protein, and its occupancy is functional (Figure 2C) (Christophorou et al., 2014). Taken together, these convergent lines of evidence point to a role of PADI4-mediated enhancer regulation.

### Deacetylation Act Cooperatively With Citrullination to Regulate Gene Expression

In addition to being a possible transcriptional target of LEF1 and pSMAD1/5, PADI4 has the potential to bind directly to, and influence the function of, LEF1 in progenitor cells as well as differentiated HF lineages. Although LEF1 is known to act as a transcriptional activator in the HF, binding of LEF1 to the histone deacetylase HDAC1 generally leads to LEF1-mediated gene repression (Billin et al., 2000).

Considering the broad HDAC1 expression pattern and general histone and non-histone deacetylase activity during hair follicle growth (Joost et al., 2020), loss of HDAC1 would likely impinge on multiple aspects of hair follicle regeneration. Indeed, loss of HDAC1 (alone, or in combination with HDAC2 to reduce redundancy), affects all epidermal lineages (LeBoeuf et al., 2010; Winter et al., 2013; Hughes et al., 2014). HDAC1 devoid hair follicles formed properly during morphogenesis but failed to enter the first resting phase (Winter et al., 2013; Hughes et al., 2014) resulting in progressive cyst formation and hair follicle atrophy.

Molecular examination revealed that both HFSC and progenitor markers were downregulated, and no hair follicle differentiation markers were expressed in the absence of HDAC1. In contrast, differentiated cysts expressed Involucrin (IVL), a marker normally associated with the epidermal lineage. Interestingly, HDAC1 binds to promoters of epidermal differentiation genes in epidermal progenitor cells (Winter et al., 2013) and upregulation of differentiation markers in the absence of HDAC1 activity correlates with increased histone acetylation levels. Ectopic expression of epidermal differentiation markers in HDAC1 devoid HF cysts suggests that HDAC1 suppresses (epidermal) lineage fate also in hair follicles.

PADI4 is described to interact with HDAC1 in several cell types (Denis et al., 2009; Nakashima et al., 2013) and gene manipulation experiments demonstrate that histone citrullination and deacetylation activity are functionally linked in regulating promoter activity. For example, PADI4 and HDAC1 simultaneously bind the pS2 promoter, resulting in acquisition of H3 arginine citrullination, loss of H3 arginine methylation and RNA polymerase II binding during the pS2 transcriptional clearing phase. In contrast, knockdown of *Padi4* and *Hdac1* by RNAi resulted in reduced H3 citrullination, increased H3 acetylation, and acquisition of H3R2 and H4R3 dimethylation marks. Interestingly, downregulation of *Hdac1* alone resulted in less pronounced epigenetic alterations, demonstrating that PADI4 and HDAC1 work cooperatively to favor citrullination and deacetylation of the pS2 promoter (Denis et al., 2009). In support of the synergistic functionality of citrullination and deacetylation protein modification, recent work demonstrate compensation on a transcriptional level – knock down of *Hdac1/2* leads to transcriptional upregulation of *Padi4*, whereas loss of *Padi4* upregulates *Hdac1/2* expression (Winter et al., 2013; Liu et al., 2018).

Given the restricted HF expression of PADI4, it is possible that PADI4/HDAC1 function as a transcriptional repressor complex to prevent aberrant expression of lineage unrelated genes in the hair follicle, potentially by interacting with lineage specific transcription factors such as LEF1. In hematopoietic stem cells, PADI4/LEF1/HDAC1 co-occupy the upstream region of the *c*-*Myc* transcriptional start site and silence gene expression *via* citrullination and deacetylation (Figure 2D) (Nakashima et al., 2013). However, in the absence of PADI4, HDAC1 fails to associate with LEF1 and *c-Myc* repression is alleviated. Considering LEF1’s known role in HS lineage specification and differentiation (Merrill et al., 2001; Adam et al., 2018), it is plausible to speculate that a PADI4/LEF1/HDAC1 complex would act to restrict hair shaft specification and/or differentiation by sequestering and functionally transforming the gene regulatory activity of LEF1. Nevertheless, the possibility of PADI4/HDAC1 co-regulation of hair shaft genes should not be ruled out. Although PADI4/HDAC1 has only been reported to repress transcription, this complex may also support gene expression in a context-dependent manner, such as in cooperation with transcriptional coactivators at hair shaft gene loci.

### PADIs as Modulators of Histone Methylation

In addition to the ability of PADI4 to contribute to the epigenetic landscape by modulating histone acetylation and DNA methylation, a growing number of studies reports functional interplay between histone arginine citrullination and methylation, either by competing for the same arginine or through citrullination-dependent methylation on lysine residues. In addition, PADI4 was the first enzyme identified participating in reversing histone mono-methylation through deamination (Cuthbert et al., 2004; Hidaka et al., 2005; Raijmakers et al., 2007; Yanming et al., 2004). Therefore, the function of PADI4 generally opposes arginine methyltransferases, such as those in the Protein arginine methyltransferases (PRMT) family (Figure 2A). Several PRMTs are expressed broadly in the regenerating hair follicle (Joost et al., 2020), where they methylate distinct arginine residues on both histone tails and transcriptional coactivators, but PRMT1 is the only family member whose function has been investigated in the skin.

PRMT1 is prominently expressed in both the HF and epidermal progenitor cells (Bao et al., 2017; Joost et al., 2020) and is required for maintaining epidermal progenitor cells by supporting the expression of proliferation genes and silencing genes associated with differentiation, either by activating H4R3me2 or by functioning as a transcriptional co-factor. Loss of PRMT1 leads to depletion of the self-renewing epidermal progenitor population due to premature differentiation. PADI4 expression in hair follicle progenitor and hair shaft lineage cells could act to counterbalance PRMT1-induced H4R3me2 marks, favoring differentiation over proliferation and thereby modulating either progenitor pool size or induction of differentiation in the hair shaft lineage.

In addition, PRMT5-mediated dimethylation of H3R2 and H3R8 act to antagonize H3K27me3 repressive mark deposited by Polycomb-repressive complex (PRC) 2 (Liu et al., 2020). Both normal and leukemic hematopoietic cells deficient in *Prmt5* gained H3K27me3 globally, resulting in downregulation of targeted genes. These observations suggest PRMT5 sustains gene expression and cell proliferation by counteracting PRC2-mediated H3K27me3 transcriptional repression in the context of hematopoietic cells. In the regenerating hair follicle, PRC-mediated H3K27me3 repression acts as a transcriptional switch, governing the differential expression of HFSC and progenitor cell genes to enforce maintenance as well as transition between stem cell and progenitor states (Lien et al., 2011). Since the catalytic activity of the PRC2 complex is PRMT5 independent and histone modifications have been shown to have an allosteric effect on histone modifiers (Moritz and Trievel, 2018), PRC2-catalyzed H3K27me3 may be impaired by the presence of H3R2me2s or H3R8me2s deposited by PRMT5. PADI4 would hence act to counteract PRMT-mediated transcriptional programs by directly antagonizing arginine dimethylation, thereby indirectly preventing ectopic activation of PRC2-silenced genes.

### Alopecia – Inflammation Mediated Hair Loss

Considering the complexities of hair follicle formation, it is perhaps surprising that hair follicles are at all able to faithfully regenerate. In addition, hair follicle regeneration is continuously challenged by injurious and infectious insults. Despite this, an infection rarely elicits systemic responses but is contained at the site of transgression, a feature likely stemming from several intricate defence mechanisms employed by the hair follicle epithelium (Christoph et al., 2000; Paus et al., 1998). Failure to respond to injury or contain infection does however, through different mechanisms, commonly lead to hair follicle loss, or alopecia. Inflammatory alopecia results in the loss of hair follicles and/or hair shafts (Bolduc et al., 2016a, b; Harries and Paus, 2010). Inflammation is mediated by an innate (neutrophilic) and adaptive (lymphocytic) cellular component and it is likely that the interface between the innate and adaptive immunity is critical in the development of alopecia (Figure 3).

Upon microbial invasion, epithelial cells in the hair follicle release antimicrobial peptides (Reithmayer et al., 2009) together with pro-inflammatory cyto- and chemokines to attract immune infiltration and clear invasion (Figure 3A). Additionally, hair follicle bulge and bulb epithelium reside in a state of relative immune privilege, expressing low levels of MHC class I as to prevent the presentation of self-antigens to autoreactive T-cells (Ito et al., 2004), while also producing immunosuppressant molecules such as TGF-β2 and cortisol (Paus et al., 2005; Anzai et al., 2019). Hair follicle stem cells also express CD200, functioning as a “no-danger” signal (Rosenblum et al., 2006) by promoting anti-inflammatory and immune-tolerance signaling to CD200R expressing T-cells and macrophages (Rosenblum et al., 2004).

Collapse of the immune privilege is a likely contributory mechanism leading to alopecia. Lesioned human scalp shows upregulation of MHC class I and II (Harries et al., 2010; Paus and Cotsarelis, 1999) and downregulation of CD200 (Rosenblum et al., 2004; Rosenblum et al., 2006) in the bulge (HFSCs), corroborated by the deletion of CD200 that in a mouse model resulted in peri-follicular immune cell infiltration and subsequently alopecia. Furthermore, pro-inflammatory cytokine INF-γ stimulation leads to MHC-I upregulation in the bulge, bulb, and matrix regions (Ito et al., 2004), potentiating a breakage of tolerance. The source of IFN-γ is predominantly activated T-cells and macrophages, emphasising the requirement of a pro-inflammatory environment preceding the lymphocytic infiltrate observed in alopecia (Figure 3G). Nonetheless, once the immune privilege is compromised, the hair follicle’s defence strategies are limited – premature initiation of catagen, dystrophic anagen (shedding of hair shaft), or replacement of epithelium with fibrous tissue – resulting in the onset of alopecia (Paus and Cotsarelis, 1999; Harries and Paus, 2010). Destructive immune assault to the bulge results in irreversible alopecia, such as Central centrifugal cicatricial alopecia (CCCA), due to depletion of the HFSC pool (Jaworsky et al., 1992; Pozdnyakova and Mahalingam, 2008; Al-Refu et al., 2009). In contrast, alopecia areata is characterized by an immune infiltrate residing in the bulb progenitor cell region (Gilhar et al., 2007; Gilhar et al., 2012), sparing HFSCs and allowing for hair follicle regeneration once inflammation resolves.

### Mutations Affecting Citrullination Linked to Alopecia

Interestingly from a citrullination point of view, a human genetic variant of *PTPN22* (*Protein Tyrosine Phosphatase Non-Receptor Type 22*) is associated with severe forms of alopecia areata (Kemp et al., 2006; Betz et al., 2008), a finding which was further corroborated by two independent genome-wide association studies, which identified susceptibility loci in *PTPN22* in alopecia areata patients (Martinez-Mir et al., 2007; Petukhova et al., 2010). Physical interaction of PTPN22 with PADI4 in immune cells sequesters PADI4 enzymatic activity, whereas PTPN22-deficiency results in hypercitrullination and a higher propensity for neutrophil activation to form neutrophil extracellular traps (NETs, discussed below) (Chang et al., 2015). Additionally, the C1858T *PTPN22* polymorphism associated with alopecia areata (Betz et al., 2008; Petukhova et al., 2010) was shown to be directly linked with the inability of PTPN22 to suppress PADI4 activity, and enhance inflammation-stimulated NET formation in rheumatoid arthritis (Chang et al., 2015). Apart from calcium, PTPN22 is thus far the only identified biological regulator of PADI4 activity.

As previously discussed, human *PADI3* mutations are associated with Central centrifugal cicatricial alopecia (CCCA), a scarring alopecia developing in scalp regions exposed to repetitive mechanical stress and as a consequence, inflammation (Malki et al., 2019). In contrast to the mechanism described for PTPN22 in directly affecting PADI4 activity in neutrophils, mutations in *PADI3* reduce the citrullinating activity in the hair follicle itself, thereby affecting protein target stability. CCCA patients display lower expression of trichohyalin and S100A3, known PADI3 targets in the hair follicle, which in turn affects the hair structure and resistance to mechanical stress of the hair shaft. Carriers of *PADI3* mutations hence display increased susceptibility to inflammation-induced hair loss due to intrinsic structural hair shaft defects caused by reduced citrullination.

### Neutrophil Activation – Role of PADI4

Neutrophils are principal effectors of the innate immune system and are the first immune cells to migrate to a site of infection or damage (Kolaczkowska and Kubes, 2013). Neutrophils act to kill microorganisms as well as modulating the immune response predominantly through the release of protein containing neutrophilic granules (Papayannopoulos, 2018), and Neutrophil Extracellular Traps (NETs), a mesh-like structure composed of decondensed chromatin containing histones and antimicrobial agents (Brinkmann et al., 2004). NETs function to physically immobilize and neutralize invading pathogens but have also been shown to contribute to the pathology of autoimmune and inflammatory diseases (Garcia-Romo et al., 2011; Knight et al., 2013; Lowes et al., 2014; Lee K. H. et al., 2017; Chiang et al., 2019; Dinallo et al., 2019; Fert-Bober et al., 2020; Yang et al., 2021).

Although citrullinated histones have long been considered a hallmark of NETs (Brinkmann et al., 2004), and PADI4 an essential driver of NET formation (Wang et al., 2009; Li et al., 2010; Hemmers et al., 2011; Lewis et al., 2015; Thiam et al., 2020), opposing studies showed that NETs could form without the presence of citrullinated histones, suggesting NET formation can be PADI4-independent (Parker et al., 2012; Konig and Andrade, 2016; Kenny et al., 2017). More recent work delineating the molecular mechanisms of NET formation revealed distinct PADI4 dependent and independent pathways generating NETs, stemming from the type of activating agent stimulating the neutrophil to generate NET (Boeltz et al., 2019; Rosazza et al., 2021).

Nevertheless, neutrophils do express high levels of PADI2 and PADI4 (Asaga et al., 2001; Darrah et al., 2012; Zhou et al., 2017), and NET-associated citrullination is highly correlated with autoinflammatory disease (Kessenbrock et al., 2009; Khandpur et al., 2013; Van Avondt and Hartl, 2018; Castanheira and Kubes, 2019) indicating that whether PADI-activity is essential for NET formation or not, citrullination contributes to NET-associated inflammation.

### Citrullination Orchestrates a Self-Perpetuating Inflammatory Environment

It is likely that even minor inflammatory events cause tissue residing macrophages or mast cells to release proinflammatory cytokines such as IL-1β, IL-6, IL-8, TNF-α and IFN-γ, which in addition to pathogen-derived ionophores and lipopolysaccharides (LPS) (Thiam et al., 2020), are able to stimulate PADI4 activity and NET formation in neutrophils (Figure 3A) (Neeli et al., 2008; Liu et al., 2017; Papayannopoulos, 2018; Schon and Erpenbeck, 2018; Klopf et al., 2021). Once stimulated, neutrophils themselves induce expression of proinflammatory IL-8 and TNF-α, further fuelling neutrophil activation and NET release in a positive feed-back loop (Figure 3B).

The expression of pro-inflammatory cytokines is known to be regulated by NF-κB signaling, a transcriptional effector whose activity is influenced by direct citrullination in immune cells, as well as indirectly by the presence of citrullinated H3 in NETs. Interestingly, LPS-induced neutrophil activation leads to endogenous NF-κB dependent expression of pro-inflammatory TNF-α and IL-1β (Figure 3C) (Sun et al., 2017). In this study, PADI4 was found to citrullinate NF-κB at four sites within the p65 Rel homology domain. Citrullination enhanced p65 interaction with Importin α3, facilitated p65 nuclear localization and augmented transcription of target genes IL-1β and TNF-α.

Additionally, Tsourouktsoglou et al. (2020) were able to generate NETs both in the presence and absence of PADI4. Fragmented nucleosomes expulsed during NET formation in neutrophils were sequestered by monocytes, bound Toll-like receptor (TLR) 4 and activated NF-κB-dependent expression of pro-inflammatory cytokines IL-1β, IL-1α, and IL-6. Interestingly, PADI4-mediated H3 citrullination increased H3 binding to TLR4 and potentiated the pro-inflammatory environment. In contrast, PADI4-deficiency reduced inflammation although NETs were still generated (Figure 3D). These proinflammatory cytokines are in turn potent activators of NF-κB expression (Shao et al., 2019), indicating the importance of self-perpetuating mechanisms in establishing a pro-inflammatory environment.

### The Antimicrobial Peptide LL-37 is Found in NETs and Contributes to Inflammation

The Cathelicidin antimicrobial peptide LL-37 is found in NETs where it binds DNA, forming an immunogenic complex insensitive to DNAse I (Pinegin et al., 2015), thereby preventing efficient chromatin clearing and contributing to the pro-inflammatory effects of NETs (Hakkim et al., 2010). LL-37 has been shown to trigger T-cell activation and subsequent IFN-γ and IL-17 production (Lande et al., 2014) as well as directly stimulate IFN-γ release in plasmacytoid dendritic cells (Lande et al., 2011), further expanding the inflammatory environment.

PADI2 and PADI4 localized to NETs readily citrullinates LL-37 (Casanova et al., 2020; Chapman et al., 2019) (Figure 3B). Citrullinated LL-37 displays impaired microbicidal effects (Al-Adwani et al., 2020; Kilsgard et al., 2012), and is unable to lower the production of IL-6 and IL-8 in virus-infected epithelial cells, (Casanova et al., 2020), suggesting that citrullinated LL-37 is less effective at decreasing an inflammatory environment. Furthermore, work in systemic lupus erythematosus (SLE) (Lande et al., 2020) demonstrate that citLL-37 acts as a stronger T cell receptor (TCR) agonist and generates a more pronounced proliferative response in T-cells at lower concentrations compared to native LL-37, suggesting that the levels of citLL-37 generated during NET release would be sufficient to induce an autoreactive T-cell response (Figure 3E). Furthermore, T cells exposed to NETs are more prone to mediating an adaptive immune response (Tillack et al., 2012), placing citrullination-associated features at the interface between innate and adaptive immunity.

### PADIs as Regulators of T-Cell Polarization and Cytokine Profile

Activated T-cells are important mediators of hair follicle destruction (Cetin et al., 2009; Bertolini et al., 2020). In addition to being targets of citrullination-dependent activation signaling emanating from neutrophils, T-cells themselves express both PADI2 and PADI4 (Liu et al., 2018; Sun et al., 2019), which functionally contribute to the inflammatory T-cell response (Figure 3E).

In a murine SLE model, the deletion of either *Padi2* or *Padi4*, or treatment with PADI-inhibitor Cl-amidine, resulted in decreased Th1 and Th17 cell numbers and production of associated cytokine IFN- γ, while Th2 cell numbers were increased (Kawalkowska et al., 2016; Liu et al., 2018), suggesting that PADI activity modulates Th subset polarization and subsequently the inflammatory cytokine profiles. Mechanistically, PADI2 skew Th polarization towards Th17 fate by simultaneous citrullination of key transcription factors GATA3 and ROR-γ-t. Citrullination of a single arginine residue in GATA3 weakens its DNA-binding abilities, whereas conversely, DNA binding capacity is enhanced by citrullination of ROR-γ-t (Figure 3F) (Sun et al., 2019), thereby balancing target gene expression and effectively Th cell fate. Interestingly, Th17 cells and associated cytokine profile have been reported to be enriched within lesions of alopecia areata and correlated with disease severity (Tanemura et al., 2013; Han et al., 2015; Loh et al., 2018). The exact involvement of Th17 cells and the contribution of citrullination within such lesions requires further investigation.

Just as activated neutrophils produce pro-inflammatory TNF-α and IL-8, stimulation of Jurkat T-cells results in increased TNF-α and IL-8 expression through a citrullination dependent mechanism. Sharma et al. showed antagonizing binding of the transcriptional repressor HP1α and PADI4 at *Tnf-α* and *Il-8* promoter regions (Sharma et al., 2012). Citrullination of H3R8 (H3Cit8) disabled binding of HP1α at H3K9me3 residues. Stimulation of the Jurkat cells resulted in increased levels of H3Cit8+K9me3 dually marked histones, and increased expression of TNF-α and IL-8, a phenomenon which was reversed by PADI4 inhibition through Cl-amidine treatment. It is possible that PADI4 antagonizes HP1α-mediated repression of *Tnf-α* and *Il-8* promoters also in activated neutrophils, however this remains to be determined.

TNF-α expression is additionally regulated by NF-κB in a citrullination dependent manner (as discussed above). However the potential synergies between histone citrullination at the *Tnf-α* promoter and direct citrullination of a TNF-α transcriptional effector remains to be investigated. What is clear is however that transcriptional activity within T-cell populations can be fine-tuned by intrinsic citrullination, positioning citrullination as a means to achieve a plethora of actions in both innate and adaptive immunity.

### Citrullination – More Than Meets the Eye

Despite an increasing number of reports delineating the protein expression of PADI enzymes, the tools used to identify PADIs and their enzymatic activity are still underdeveloped, adding up to a discordant and sometimes even contradictory picture. Here we have chosen to rely predominantly on mRNA expression for detailing cell populations that express PADI enzymes in the hair follicle and inflammation. In cases where protein characterization is available, mRNA and protein expression profiles largely overlap. However challenging to visualize, there is reason to believe that the function of these elusive proteins is far more complex than first perceived. Our current understanding of citrullination suggests that the functional outcome of citrullination is context dependent and dynamically regulated. The deiminase activity of PADIs is thus employed by most cells, in one way or the other, to achieve an array of functions.

In view of citrullination as a stable, and possibly irreversible, modification it is intuitive to localize PADIs to differentiated IRS and medulla lineages, where citrullination acts to mediate structural rigidity of the hair shaft. However, citrullinated proteins are also linked to progenitor proliferation and immune cell activation, cell types continuously responding to changing environmental cues, indicating that citrullination can be used by cells in a more multifaceted way than so far appreciated. Functionally, citrullination is seldom an end in itself, but rather acts in conjunction with other types of posttranslational modifications, be it on histones or otherwise. As such, citrullination seems to be a precision tool used to fine-tune the molecular functionalities a cell requires to maintain the complex interplay of protein and gene regulation, normally and in disease.

Recent findings have unveiled the broad implications PADIs may have on stem cell maintenance and differentiation in diverse stem cell niches. In the HFSC lineage, PADI expression correlate with HFSC activation, progenitor specification and hair shaft lineage differentiation, positioning PADIs at key HF lineage transition points. Whereas PADI1 and PADI3 in the regenerating hair follicle are currently exclusively associated with citrullination of structural hair proteins, work detailing the PADI1 and PADI3 HF cell state specific citrullinomes would likely provide new insights into the HF specific role of PADI1/3, in differentiating as well as self-renewing cell populations. Taking into account the redundancy of PADI1 and PADI3 in the HF, it is likely that establishment of a mouse line genetically devoid of both PADI1/3 expression would be required to grasp the full functional magnitude of these enzymes in the regenerating hair follicle.

In contrast to the relatively narrow functionality linked to PADI1 and PADI3, the implications of PADI4 expression in the hair follicle cast a wider web. PADI4 has a known role in regulating stem cell renewal and specification in stem cell lineages, and expression in activated HFSCs and progenitor cells suggests PADI4 could act analogously in HFs. The ability to citrullinate DNA methylases, modulate the function of key HF lineage determining transcription factors, synergizing with histone deacetylases and antagonizing arginine methylases indicates that the action of PADI4 in the regenerating hair follicle could be complex and modulate all stages of regeneration. Although aspects of epigenetic regulation of HFSC lineage progression is well established, how citrullination impinges on the HF epigenetic landscape is completely unknown. Delineating potential PADI4 dependent fine-tuning of the epigenetic landscape suggest an overall role in maintaining self-renewal and restricting differentiation.

Considering the complex function of PADIs in the HFSC lineage, it is perhaps not surprising that PADIs target a variety of pro-inflammatory signaling pathways in both neutrophils and T-cells in the skin. Paradoxically, citrullination of cytokines associated with neutrophil-mediated inflammation such as IL-8, CXCL-10, CXCL-11 and CXCL-12 reduces their respective inflammatory potency (Loos et al., 2008; Proost et al., 2008; Struyf et al., 2009). Moreover, citrullinated TNF-α show reduced ability to stimulate production of inflammatory cytokines by fibroblasts *in vitro* (Moelants et al., 2013). However tempting to speculate that citrullination, in addition to its established pro-inflammatory role could act as an inflammatory antagonist, citrullination of these chemokines were investigated *in vitro* without addressing the source of PADIs or the involvement of NETs. It therefore remains to be determined if chemokine citrullination occurs in an attempt to negatively balance the inflammatory environment initially employed to attract and activate neutrophils and NET formation.

Although the lion’s share of citrullination-dependent inflammatory events leading up to hair follicle stem cell destruction in alopecia is mediated through immune cells, it is likely that alterations in epithelial PADI expression contributes to disease progression. NF-κB was shown to bind an intronic enhancer element of *PADI1* and was found to be crucial for its expression in human keratinocytes (Ying et al., 2010). Although evidence suggests that PADI1 activity is diminished in psoriatic skin (Ishida-Yamamoto et al., 2000), and IL-22 driven hyperproliferation in psoriatic keratinocytes downregulate PADI1 (Padhi et al., 2021), it is enthralling to envision an epithelial interdependency between PADI enzymes and NF-κB signaling, especially given the broad interconnectedness these have been shown to display in immune cells.

Additionally, it is noteworthy that human *PTPN22* mutations associated with alopecia areata and rheumatoid arthritis, also negatively regulates lymphocyte activation by reducing the PTPN22 phosphatase activity (Wu, 2006). Although PTPN22 inhibition of PADI4 activity is phosphorylation-independent, removal of either mode of PTPN22 suppressive abilities, be it *via* TCR signaling by phosphorylation or physical interaction with PADI4, both lead to immune cell activation. It would be of interest to investigate if the blockage of PTPN22 phosphorylation (and in this way activation of TCR signaling) would correlate to citrullination within the regulatory transcriptional landscape brought about by TCR signaling, either *via* citrullination of transcription factors or histones.

Given the contribution of PADIs to autoinflammatory diseases such as rheumatoid arthritis, systemic lupus erythematosus and psoriasis, it is likely that the pathogenesis of alopecia follows similar disease trajectories. Both immune cell involvement and the cytokine profile driving the pro-inflammatory environment in alopecia match those observed in citrullination-associated autoimmune diseases. Although there are obvious gaps in our understanding of how PADIs directly and indirectly influence hair follicle regeneration normally and during inflammatory alopecia, drawing from other stem cell niches or inflammatory diseases enables identification of common mechanisms likely applicable to the hair follicle. Continuous technological and methodological development will undoubtedly further our understanding of citrullination in the skin, normally and during inflammatory disease.

## References

[B1] Adam R. C. Yang H. Ge Y. Lien W.-H. Wang P. Zhao Y. (2018). Temporal Layering of Signaling Effectors Drives Chromatin Remodeling during Hair Follicle Stem Cell Lineage Progression. Cell stem cell 22, 398–413. e397. 10.1016/j.stem.2017.12.004 29337183 PMC6425486

[B2] Adam R. C. Yang H. Rockowitz S. Larsen S. B. Nikolova M. Oristian D. S. (2015). Pioneer Factors Govern Super-enhancer Dynamics in Stem Cell Plasticity and Lineage Choice. Nature 521, 366–370. 10.1038/nature14289 25799994 PMC4482136

[B3] Al-Adwani S. Wallin C. Balhuizen M. D. Veldhuizen E. J. A. Coorens M. Landreh M. (2020). Studies on Citrullinated LL-37: Detection in Human Airways, Antibacterial Effects and Biophysical Properties. Sci. Rep. 10, 2376. 10.1038/s41598-020-59071-7 32047184 PMC7012854

[B4] Al-Refu K. Edward S. Ingham E. Goodfield M. (2009). Expression of Hair Follicle Stem Cells Detected by Cytokeratin 15 Stain: Implications for Pathogenesis of the Scarring Process in Cutaneous Lupus Erythematosus. Br. J. Dermatol. 160, 1188–1196. 10.1111/j.1365-2133.2009.09074.x 19298282

[B5] Anzai A. Wang E. H. C. Lee E. Y. Aoki V. Christiano A. M. (2019). Pathomechanisms of Immune-Mediated Alopecia. Int. Immunol. 31, 439–447. 10.1093/intimm/dxz039 31050755 PMC6940981

[B6] Arita K. Hashimoto H. Shimizu T. Nakashima K. Yamada M. Sato M. (2004). Structural Basis for Ca2+-Induced Activation of Human PAD4. Nat. Struct. Mol. Biol. 11, 777–783. 10.1038/nsmb799 15247907

[B7] Asaga H. Nakashima K. Senshu T. Ishigami A. Yamada M. (2001). Immunocytochemical Localization of Peptidylarginine Deiminase in Human Eosinophils and Neutrophils. J. Leukoc. Biol. 70, 46–51. 10.1189/jlb.70.1.46 11435484

[B8] Bao X. Siprashvili Z. Zarnegar B. J. Shenoy R. M. Rios E. J. Nady N. (2017). CSNK1a1 Regulates PRMT1 to Maintain the Progenitor State in Self-Renewing Somatic Tissue. Dev. Cel. 43, 227–239. e225. 10.1016/j.devcel.2017.08.021 PMC5659279 28943242

[B9] Bertolini M. McElwee K. Gilhar A. Bulfone‐Paus S. Paus R. (2020). Hair Follicle Immune Privilege and its Collapse in Alopecia Areata. Exp. Dermatol. 29, 703–725. 10.1111/exd.14155 32682334

[B10] Betz R. C. König K. Flaquer A. Redler S. Eigelshoven S. Kortüm A. K. (2008). The R620W Polymorphism in PTPN22 Confers General Susceptibility for the Development of Alopecia Areata. Br. J. Dermatol. 158, 389–391. 10.1111/j.1365-2133.2007.08312.x 18028494

[B11] Billin A. N. Thirlwell H. Ayer D. E. (2000). β-Catenin-Histone Deacetylase Interactions Regulate the Transition of LEF1 from a Transcriptional Repressor to an Activator. Mol. Cel Biol 20, 6882–6890. 10.1128/mcb.20.18.6882-6890.2000 PMC88764 10958684

[B12] Bock C. Beerman I. Lien W.-H. Smith Z. D. Gu H. Boyle P. (2012). DNA Methylation Dynamics during *In Vivo* Differentiation of Blood and Skin Stem Cells. Mol. Cel 47, 633–647. 10.1016/j.molcel.2012.06.019 PMC3428428 22841485

[B13] Boeltz S. Amini P. Anders H.-J. Andrade F. Bilyy R. Chatfield S. (2019). To NET or Not to NET:current Opinions and State of the Science Regarding the Formation of Neutrophil Extracellular Traps. Cell Death Differ 26, 395–408. 10.1038/s41418-018-0261-x 30622307 PMC6370810

[B14] Bolduc C. Sperling L. C. Shapiro J. (2016a). Primary Cicatricial Alopecia. J. Am. Acad. Dermatol. 75, 1081–1099. 10.1016/j.jaad.2014.09.058 27846944

[B15] Bolduc C. Sperling L. C. Shapiro J. (2016b). Primary Cicatricial Alopecia. J. Am. Acad. Dermatol. 75, 1101–1117. 10.1016/j.jaad.2015.01.056 27846945

[B16] Brinkmann V. Reichard U. Goosmann C. Fauler B. Uhlemann Y. Weiss D. S. (2004). Neutrophil Extracellular Traps Kill Bacteria. Science 303, 1532–1535. 10.1126/science.1092385 15001782

[B17] Briot J. Simon M. Méchin M. C. (2020). Deimination, Intermediate Filaments and Associated Proteins. Int. J. Mol. Sci. 21. 10.3390/ijms21228746 PMC7699402 33228136

[B18] Casanova V. Sousa F. H. Shakamuri P. Svoboda P. Buch C. D'Acremont M. (2020). Citrullination Alters the Antiviral and Immunomodulatory Activities of the Human Cathelicidin LL-37 during Rhinovirus Infection. Front. Immunol. 11, 85. 10.3389/fimmu.2020.00085 32117246 PMC7010803

[B19] Castanheira F. V. S. Kubes P. (2019). Neutrophils and NETs in Modulating Acute and Chronic Inflammation. Blood 133, 2178–2185. 10.1182/blood-2018-11-844530 30898862

[B20] Cau L. Méchin M.-C. Simon M. (2018). Peptidylarginine Deiminases and Deiminated Proteins at the Epidermal Barrier. Exp. Dermatol. 27, 852–858. 10.1111/exd.13684 29756256

[B21] Cetin E. D. Şavk E. Uslu M. Eskin M. Karul A. (2009). Investigation of the Inflammatory Mechanisms in Alopecia Areata. Am. J. Dermatopathol 31, 53–60. 10.1097/dad.0b013e318185a66e 19155726

[B22] Chang H.-H. Dwivedi N. Nicholas A. P. Ho I.-C. (2015). The W620 Polymorphism in PTPN22 Disrupts its Interaction with Peptidylarginine Deiminase Type 4 and Enhances Citrullination and NETosis. Arthritis Rheumatol. 67, 2323–2334. 10.1002/art.39215 26019128

[B23] Chapman E. A. Lyon M. Simpson D. Mason D. Beynon R. J. Moots R. J. (2019). Caught in a Trap? Proteomic Analysis of Neutrophil Extracellular Traps in Rheumatoid Arthritis and Systemic Lupus Erythematosus. Front. Immunol. 10, 423. 10.3389/fimmu.2019.00423 30915077 PMC6421309

[B24] Chen D. Y. Ferguson I. M. Braun K. A. Sutton L. A. Helton N. M. Ramakrishnan S. M. (2021). Dnmt3a Deficiency in the Skin Causes Focal, Canonical DNA Hypomethylation and a Cellular Proliferation Phenotype. Proc. Natl. Acad. Sci. U S A. 118, e2022760118. 10.1073/pnas.2022760118 33846253 PMC8072215

[B25] Chiang C.-C. Cheng W.-J. Korinek M. Lin C.-Y. Hwang T.-L. (2019). Neutrophils in Psoriasis. Front. Immunol. 10, 2376. 10.3389/fimmu.2019.02376 31649677 PMC6794444

[B26] Christoph T. Müller-Röver S. Audring H. Tobin D. J. Hermes B. Cotsarelis G. (2000). The Human Hair Follicle Immune System: Cellular Composition and Immune Privilege. Br. J. Dermatol. 142, 862–873. 10.1046/j.1365-2133.2000.03464.x 10809841

[B27] Christophorou M. A. Castelo-Branco G. Halley-Stott R. P. Oliveira C. S. Loos R. Radzisheuskaya A. (2014). Citrullination Regulates Pluripotency and Histone H1 Binding to Chromatin. Nature 507, 104–108. 10.1038/nature12942 24463520 PMC4843970

[B28] Cuthbert G. L. Daujat S. Snowden A. W. Erdjument-Bromage H. Hagiwara T. Yamada M. (2004). Histone Deimination Antagonizes Arginine Methylation. Cell 118, 545–553. 10.1016/j.cell.2004.08.020 15339660

[B29] Darrah E. Rosen A. Giles J. T. Andrade F. (2012). Peptidylarginine Deiminase 2, 3 and 4 Have Distinct Specificities against Cellular Substrates: Novel Insights into Autoantigen Selection in Rheumatoid Arthritis. Ann. Rheum. Dis. 71, 92–98. 10.1136/ard.2011.151712 21859690 PMC3302156

[B30] Denis H. Deplus R. Putmans P. Yamada M. Métivier R. Fuks F. (2009). Functional Connection between Deimination and Deacetylation of Histones. Mol. Cel Biol 29, 4982–4993. 10.1128/mcb.00285-09 PMC2738279 19581286

[B31] Deplus R. Denis H. Putmans P. Calonne E. Fourrez M. Yamamoto K. (2014). Citrullination of DNMT3A by PADI4 Regulates its Stability and Controls DNA Methylation. Nucleic Acids Res. 42, 8285–8296. 10.1093/nar/gku522 24957603 PMC4117755

[B32] Dinallo V. Marafini I. Di Fusco D. Laudisi F. Franzè E. Di Grazia A. (2019). Neutrophil Extracellular Traps Sustain Inflammatory Signals in Ulcerative Colitis. J. Crohns Colitis 13, 772–784. 10.1093/ecco-jcc/jjy215 30715224

[B33] Falcão A. M. Meijer M. Scaglione A. Rinwa P. Agirre E. Liang J. (2019). PAD2-Mediated Citrullination Contributes to Efficient Oligodendrocyte Differentiation and Myelination. Cel Rep. 27, 1090–1102. 10.1016/j.celrep.2019.03.108 PMC6486480 31018126

[B34] Fert-Bober J. Darrah E. Andrade F. (2020). Insights into the Study and Origin of the Citrullinome in Rheumatoid Arthritis. Immunol. Rev. 294, 133–147. 10.1111/imr.12834 31876028 PMC8061312

[B35] Garcia-Romo G. S. Caielli S. Vega B. Connolly J. Allantaz F. Xu Z. (2011). Netting Neutrophils Are Major Inducers of Type I IFN Production in Pediatric Systemic Lupus Erythematosus. Sci. Transl Med. 3, 73ra20. 10.1126/scitranslmed.3001201 PMC3143837 21389264

[B36] Genander M. Cook P. J. Ramsköld D. Keyes B. E. Mertz A. F. Sandberg R. (2014). BMP Signaling and its pSMAD1/5 Target Genes Differentially Regulate Hair Follicle Stem Cell Lineages. Cell Stem Cell 15, 619–633. 10.1016/j.stem.2014.09.009 25312496 PMC4276600

[B37] Ghari F. Quirke A. M. Munro S. Kawalkowska J. Picaud S. McGouran J. (2016). Citrullination-acetylation Interplay Guides E2F-1 Activity during the Inflammatory Response. Sci. Adv. 2, e1501257. 10.1126/sciadv.1501257 26989780 PMC4788482

[B38] Gilhar A. Etzioni A. Paus R. (2012). Alopecia Areata. N. Engl. J. Med. 366, 1515–1525. 10.1056/nejmra1103442 22512484

[B39] Gilhar A. Paus R. Kalish R. S. (2007). Lymphocytes, Neuropeptides, and Genes Involved in Alopecia Areata. J. Clin. Invest. 117, 2019–2027. 10.1172/jci31942 17671634 PMC1934574

[B40] Greco V. Chen T. Rendl M. Schober M. Pasolli H. A. Stokes N. (2009). A Two-step Mechanism for Stem Cell Activation during Hair Regeneration. Cell Stem Cell 4, 155–169. 10.1016/j.stem.2008.12.009 19200804 PMC2668200

[B41] Guertin M. J. Zhang X. Anguish L. Kim S. Varticovski L. Lis J. T. (2014). Targeted H3R26 Deimination Specifically Facilitates Estrogen Receptor Binding by Modifying Nucleosome Structure. Plos Genet. 10, e1004613. 10.1371/journal.pgen.1004613 25211228 PMC4161307

[B42] Guo Q. Fast W. (2011). Citrullination of Inhibitor of Growth 4 (ING4) by Peptidylarginine Deminase 4 (PAD4) Disrupts the Interaction between ING4 and P53. J. Biol. Chem. 286, 17069–17078. 10.1074/jbc.m111.230961 21454715 PMC3089551

[B43] Hagiwara T. Hidaka Y. Yamada M. (2005). Deimination of Histone H2A and H4 at Arginine 3 in HL-60 Granulocytes. Biochemistry 44, 5827–5834. 10.1021/bi047505c 15823041

[B44] Hakkim A. Furnrohr B. G. Amann K. Laube B. Abed U. A. Brinkmann V. (2010). Impairment of Neutrophil Extracellular Trap Degradation Is Associated with Lupus Nephritis. Proc. Natl. Acad. Sci. 107, 9813–9818. 10.1073/pnas.0909927107 20439745 PMC2906830

[B45] Han Y.-M. Sheng Y.-Y. Xu F. Qi S.-S. Liu X.-J. Hu R.-M. (2015). Imbalance of T-Helper 17 and Regulatory T Cells in Patients with Alopecia Areata. J. Dermatol. 42, 981–988. 10.1111/1346-8138.12978 26077574

[B46] Harries M. J. Meyer K. C. Chaudhry I. H. Griffiths C. E. Paus R. (2010). Does Collapse of Immune Privilege in the Hair-Follicle Bulge Play a Role in the Pathogenesis of Primary Cicatricial Alopecia. Clin. Exp. Dermatol. 35, 637–644. 10.1111/j.1365-2230.2009.03692.x 19886964

[B47] Harries M. J. Paus R. (2010). The Pathogenesis of Primary Cicatricial Alopecias. Am. J. Pathol. 177, 2152–2162. 10.2353/ajpath.2010.100454 20889564 PMC2966773

[B48] Hemmers S. Teijaro J. R. Arandjelovic S. Mowen K. A. (2011). PAD4-mediated Neutrophil Extracellular Trap Formation Is Not Required for Immunity against Influenza Infection. PLoS One 6, e22043. 10.1371/journal.pone.0022043 21779371 PMC3133614

[B49] Hidaka Y. Hagiwara T. Yamada M. (2005). Methylation of the Guanidino Group of Arginine Residues Prevents Citrullination by Peptidylarginine Deiminase IV. FEBS Lett. 579, 4088–4092. 10.1016/j.febslet.2005.06.035 16023115

[B50] Hu L. Bikle D. D. Oda Y. (2014). Reciprocal Role of Vitamin D Receptor on β-catenin Regulated Keratinocyte Proliferation and Differentiation. J. Steroid Biochem. Mol. Biol. 144, 237–241. 10.1016/j.jsbmb.2013.11.002 24239508 PMC4061268

[B51] Hughes M. W. Jiang T.-X. Lin S.-J. Leung Y. Kobielak K. Widelitz R. B. (2014). Disrupted Ectodermal Organ Morphogenesis in Mice with a Conditional Histone Deacetylase 1, 2 Deletion in the Epidermis. J. Invest. Dermatol. 134, 24–32. 10.1038/jid.2013.283 23792463 PMC3843967

[B52] Ishida-Yamamoto A. Takahashi H. Iizuka H. Senshu T. Akiyama K. Nomura K. (2000). Decreased Deiminated Keratin K1 in Psoriatic Hyperproliferative Epidermis. J. Invest. Dermatol. 114, 701–705. 10.1046/j.1523-1747.2000.00936.x 10733676

[B53] Ito T. Ito N. Bettermann A. Tokura Y. Takigawa M. Paus R. (2004). Collapse and Restoration of MHC Class-I-dependent Immune Privilege. Am. J. Pathol. 164, 623–634. 10.1016/s0002-9440(10)63151-3 14742267 PMC1602279

[B54] Jaworsky C. Kligman A. M. Murphy G. F. (1992). Characterization of Inflammatory Infiltrates in Male Pattern Alopecia: Implications for Pathogenesis. Br. J. Dermatol. 127, 239–246. 10.1111/j.1365-2133.1992.tb00121.x 1390168

[B55] Joost S. Annusver K. Jacob T. Sun X. Dalessandri T. Sivan U. (2020). The Molecular Anatomy of Mouse Skin during Hair Growth and Rest. Cell Stem Cell 26, 441–457. 10.1016/j.stem.2020.01.012 32109378

[B56] Kan R. Jin M. Subramanian V. Causey C. P. Thompson P. R. Coonrod S. A. (2012). Potential Role for PADI-Mediated Histone Citrullination in Preimplantation Development. BMC Dev. Biol. 12, 19. 10.1186/1471-213x-12-19 22712504 PMC3430579

[B57] Kaufman C. K. Zhou P. Amalia Pasolli H. Rendl M. Bolotin D. Lim K.-C. (2003). GATA-3: an Unexpected Regulator of Cell Lineage Determination in Skin. Genes Dev. 17, 2108–2122. 10.1101/gad.1115203 12923059 PMC196453

[B58] Kawalkowska J. Quirke A.-M. Ghari F. Davis S. Subramanian V. Thompson P. R. (2016). Abrogation of Collagen-Induced Arthritis by a Peptidyl Arginine Deiminase Inhibitor Is Associated with Modulation of T Cell-Mediated Immune Responses. Sci. Rep. 6, 26430. 10.1038/srep26430 27210478 PMC4876390

[B59] Kemp E. H. McDonagh A. J. G. Wengraf D. A. Messenger A. G. Gawkrodger D. J. Cork M. J. (2006). The Non-synonymous C1858T Substitution in the PTPN22 Gene Is Associated with Susceptibility to the Severe Forms of Alopecia Areata. Hum. Immunol. 67, 535–539. 10.1016/j.humimm.2006.04.006 16829308

[B60] Kenny E. F. Herzig A. Krüger R. Muth A. Mondal S. Thompson P. R. (2017). Diverse Stimuli Engage Different Neutrophil Extracellular Trap Pathways. Elife 6, e24437. 10.7554/eLife.24437 28574339 PMC5496738

[B61] Kessenbrock K. Krumbholz M. Schönermarck U. Back W. Gross W. L. Werb Z. (2009). Netting Neutrophils in Autoimmune Small-Vessel Vasculitis. Nat. Med. 15, 623–625. 10.1038/nm.1959 19448636 PMC2760083

[B62] Khandpur R. Carmona-Rivera C. Vivekanandan-Giri A. Gizinski A. Yalavarthi S. Knight J. S. (2013). NETs Are a Source of Citrullinated Autoantigens and Stimulate Inflammatory Responses in Rheumatoid Arthritis. Sci. Transl Med. 5, 178ra40. 10.1126/scitranslmed.3005580 PMC3727661 23536012

[B63] Kilsgård O. Andersson P. Malmsten M. Nordin S. L. Linge H. M. Eliasson M. (2012). Peptidylarginine Deiminases Present in the Airways during Tobacco Smoking and Inflammation Can Citrullinate the Host Defense Peptide LL-37, Resulting in Altered Activities. Am. J. Respir. Cel Mol Biol 46, 240–248. 10.1165/rcmb.2010-0500oc 21960546

[B64] Kizawa K. Takahara H. Troxler H. Kleinert P. Mochida U. Heizmann C. W. (2008). Specific Citrullination Causes Assembly of a Globular S100A3 Homotetramer. J. Biol. Chem. 283, 5004–5013. 10.1074/jbc.m709357200 18083705

[B65] Klopf J. Brostjan C. Eilenberg W. Neumayer C. (2021). Neutrophil Extracellular Traps and Their Implications in Cardiovascular and Inflammatory Disease. Int. J. Mol. Sci. 22, 559. 10.3390/ijms22020559 PMC7828090 33429925

[B66] Knight J. S. Zhao W. Luo W. Subramanian V. O’Dell A. A. Yalavarthi S. (2013). Peptidylarginine Deiminase Inhibition Is Immunomodulatory and Vasculoprotective in Murine Lupus. J. Clin. Invest. 123, 2981–2993. 10.1172/jci67390 23722903 PMC3696545

[B67] Knuckley B. Causey C. P. Jones J. E. Bhatia M. Dreyton C. J. Osborne T. C. (2010). Substrate Specificity and Kinetic Studies of PADs 1, 3, and 4 Identify Potent and Selective Inhibitors of Protein Arginine Deiminase 3. Biochemistry 49, 4852–4863. 10.1021/bi100363t 20469888 PMC2884139

[B68] Kolaczkowska E. Kubes P. (2013). Neutrophil Recruitment and Function in Health and Inflammation. Nat. Rev. Immunol. 13, 159–175. 10.1038/nri3399 23435331

[B69] Kolodziej S. Kuvardina O. N. Oellerich T. Herglotz J. Backert I. Kohrs N. (2014). PADI4 Acts as a Coactivator of Tal1 by Counteracting Repressive Histone Arginine Methylation. Nat. Commun. 5, 3995. 10.1038/ncomms4995 24874575 PMC4050257

[B70] Konig M. F. Andrade F. (2016). A Critical Reappraisal of Neutrophil Extracellular Traps and NETosis Mimics Based on Differential Requirements for Protein Citrullination. Front. Immunol. 7, 461. 10.3389/fimmu.2016.00461 27867381 PMC5095114

[B71] Lai N.-S. Yu H.-C. Tung C.-H. Huang K.-Y. Huang H.-B. Lu M.-C. (2019). Increased Peptidylarginine Deiminases Expression during the Macrophage Differentiation and Participated Inflammatory Responses. Arthritis Res. Ther. 21, 108. 10.1186/s13075-019-1896-9 31039829 PMC6492328

[B72] Lande R. Ganguly D. Facchinetti V. Frasca L. Conrad C. Gregorio J. (2011). Neutrophils Activate Plasmacytoid Dendritic Cells by Releasing Self-DNA-Peptide Complexes in Systemic Lupus Erythematosus. Sci. Transl Med. 3, 73ra19. 10.1126/scitranslmed.3001180 PMC3399524 21389263

[B73] Lande R. Botti E. Jandus C. Dojcinovic D. Fanelli G. Conrad C. (2014). The Antimicrobial Peptide LL37 Is a T-Cell Autoantigen in Psoriasis. Nat. Commun. 5, 5621. 10.1038/ncomms6621 25470744

[B74] Lande R. Palazzo R. Gestermann N. Jandus C. Falchi M. Spadaro F. (2020). Native/citrullinated LL37-specific T-Cells Help Autoantibody Production in Systemic Lupus Erythematosus. Sci. Rep. 10, 5851. 10.1038/s41598-020-62480-3 32245990 PMC7125190

[B75] LeBoeuf M. Terrell A. Trivedi S. Sinha S. Epstein J. A. Olson E. N. (2010). Hdac1 and Hdac2 Act Redundantly to Control P63 and P53 Functions in Epidermal Progenitor Cells. Dev. Cel. 19, 807–818. 10.1016/j.devcel.2010.10.015 PMC3003338 21093383

[B76] Lee C.-Y. Lin C.-C. Liu Y.-L. Liu G.-Y. Liu J.-H. Hung H.-C. (2017a). Molecular Interplay between the Dimer Interface and the Substrate-Binding Site of Human Peptidylarginine Deiminase 4. Sci. Rep. 7, 42662. 10.1038/srep42662 28209966 PMC5314407

[B77] Lee C.-Y. Wang D. Wilhelm M. Zolg D. P. Schmidt T. Schnatbaum K. (2018). Mining the Human Tissue Proteome for Protein Citrullination. Mol. Cell Proteomics 17, 1378–1391. 10.1074/mcp.ra118.000696 29610271 PMC6030718

[B78] Lee K. H. Kronbichler A. Park D. D.-Y. Park Y. Moon H. Kim H. (2017b). Neutrophil Extracellular Traps (NETs) in Autoimmune Diseases: A Comprehensive Review. Autoimmun. Rev. 16, 1160–1173. 10.1016/j.autrev.2017.09.012 28899799

[B79] Lee Y.-H. Coonrod S. A. Kraus W. L. Jelinek M. A. Stallcup M. R. (2005). Regulation of Coactivator Complex Assembly and Function by Protein Arginine Methylation and Demethylimination. Proc. Natl. Acad. Sci. 102, 3611–3616. 10.1073/pnas.0407159102 15731352 PMC553305

[B80] Lewallen D. M. Bicker K. L. Subramanian V. Clancy K. W. Slade D. J. Martell J. (2015). Chemical Proteomic Platform to Identify Citrullinated Proteins. ACS Chem. Biol. 10, 2520–2528. 10.1021/acschembio.5b00438 26360112 PMC4729336

[B81] Lewis H. D. Liddle J. Coote J. E. Atkinson S. J. Barker M. D. Bax B. D. (2015). Inhibition of PAD4 Activity Is Sufficient to Disrupt Mouse and Human NET Formation. Nat. Chem. Biol. 11, 189–191. 10.1038/nchembio.1735 25622091 PMC4397581

[B82] Li P. Li M. Lindberg M. R. Kennett M. J. Xiong N. Wang Y. (2010). PAD4 Is Essential for Antibacterial Innate Immunity Mediated by Neutrophil Extracellular Traps. J. Exp. Med. 207, 1853–1862. 10.1084/jem.20100239 20733033 PMC2931169

[B83] Lien W.-H. Guo X. Polak L. Lawton L. N. Young R. A. Zheng D. (2011). Genome-wide Maps of Histone Modifications Unwind *In Vivo* Chromatin States of the Hair Follicle Lineage. Cell stem cell 9, 219–232. 10.1016/j.stem.2011.07.015 21885018 PMC3166618

[B84] Liu F. Xu Y. Lu X. Hamard P.-J. Karl D. L. Man N. (2020). PRMT5-mediated Histone Arginine Methylation Antagonizes Transcriptional Repression by Polycomb Complex PRC2. Nucleic Acids Res. 48, 2956–2968. 10.1093/nar/gkaa065 32025719 PMC7102951

[B85] Liu T. Zhang L. Joo D. Sun S. C. (2017). NF-κB Signaling in Inflammation. Signal. Transduct Target. Ther. 2, 17023. 10.1038/sigtrans.2017.23 29158945 PMC5661633

[B86] Liu Y. Lightfoot Y. L. Seto N. Carmona-Rivera C. Moore E. Goel R. (2018). Peptidylarginine Deiminases 2 and 4 Modulate Innate and Adaptive Immune Responses in TLR-7-dependent Lupus. JCI Insight 3, e124729. 10.1172/jci.insight.124729 PMC6328098 30518690

[B87] Loh S.-H. Moon H.-N. Lew B.-L. Sim W.-Y. (2018). Role of T Helper 17 Cells and T Regulatory Cells in Alopecia Areata: Comparison of Lesion and Serum Cytokine between Controls and Patients. J. Eur. Acad. Dermatol. Venereol. 32, 1028–1033. 10.1111/jdv.14775 29283462

[B88] Loos T. Mortier A. Gouwy M. Ronsse I. Put W. Lenaerts J.-P. (2008). Citrullination of CXCL10 and CXCL11 by Peptidylarginine Deiminase: a Naturally Occurring Posttranslational Modification of Chemokines and New Dimension of Immunoregulation. Blood 112, 2648–2656. 10.1182/blood-2008-04-149039 18645041

[B89] Lowes M. A. Suárez-Fariñas M. Krueger J. G. (2014). Immunology of Psoriasis. Annu. Rev. Immunol. 32, 227–255. 10.1146/annurev-immunol-032713-120225 24655295 PMC4229247

[B90] Malki L. Sarig O. Romano M.-T. Méchin M.-C. Peled A. Pavlovsky M. (2019). Variant PADI3 in Central Centrifugal Cicatricial Alopecia. N. Engl. J. Med. 380, 833–841. 10.1056/nejmoa1816614 30763140

[B91] Martinez-Mir A. Zlotogorski A. Gordon D. Petukhova L. Mo J. Gilliam T. C. (2007). Genomewide Scan for Linkage Reveals Evidence of Several Susceptibility Loci for Alopecia Areata. Am. J. Hum. Genet. 80, 316–328. 10.1086/511442 17236136 PMC1785354

[B92] Méchin M.-C. Sebbag M. Arnaud J. Nachat R. Foulquier C. Adoue V. (2007). Update on Peptidylarginine Deiminases and Deimination in Skin Physiology and Severe Human Diseases. Int. J. Cosmet. Sci. 29, 147–168. 10.1111/j.1467-2494.2007.00377.x 18489346

[B93] Méchin M. C. Takahara H. Simon M. (2020). Deimination and Peptidylarginine Deiminases in Skin Physiology and Diseases. Int. J. Mol. Sci. 21, 566. 10.3390/ijms21020566 PMC7014782 31952341

[B94] Merrill B. J. Gat U. DasGupta R. Fuchs E. (2001). Tcf3 and Lef1 Regulate Lineage Differentiation of Multipotent Stem Cells in Skin. Genes Dev. 15, 1688–1705. 10.1101/gad.891401 11445543 PMC312726

[B95] Moelants E. A. V. Mortier A. Grauwen K. Ronsse I. Van Damme J. Proost P. (2013). Citrullination of TNF-α by Peptidylarginine Deiminases Reduces its Capacity to Stimulate the Production of Inflammatory Chemokines. Cytokine 61, 161–167. 10.1016/j.cyto.2012.09.011 23075670

[B96] Moritz L. E. Trievel R. C. (2018). Structure, Mechanism, and Regulation of Polycomb-Repressive Complex 2. J. Biol. Chem. 293, 13805–13814. 10.1074/jbc.r117.800367 28912274 PMC6130931

[B97] Nachat R. Méchin M.-C. Charveron M. Serre G. Constans J. Simon M. (2005). Peptidylarginine Deiminase Isoforms Are Differentially Expressed in the Anagen Hair Follicles and Other Human Skin Appendages. J. Invest. Dermatol. 125, 34–41. 10.1111/j.0022-202x.2005.23763.x 15982300

[B98] Nakashima K. Arai S. Suzuki A. Nariai Y. Urano T. Nakayama M. (2013). PAD4 Regulates Proliferation of Multipotent Haematopoietic Cells by Controlling C-Myc Expression. Nat. Commun. 4, 1836. 10.1038/ncomms2862 23673621 PMC3674250

[B99] Neeli I. Khan S. N. Radic M. (2008). Histone Deimination as a Response to Inflammatory Stimuli in Neutrophils. J. Immunol. 180, 1895–1902. 10.4049/jimmunol.180.3.1895 18209087

[B100] Padhi A. Srivastava A. Ramesh A. Ehrstrom M. Simon M. Sonkoly E. (2021). IL-22 Downregulates Peptidylarginine Deiminase-1 in Human Keratinocytes: Adding Another Piece to the IL-22 Puzzle in Epidermal Barrier Formation. J. Invest. Dermatol. S0022-202X (21), 01658–01664. 10.1016/j.jid.2021.07.155 34352263

[B101] Pálmer H. G. Anjos-Afonso F. Carmeliet G. Takeda H. Watt F. M. (2008). The Vitamin D Receptor Is a Wnt Effector that Controls Hair Follicle Differentiation and Specifies Tumor Type in Adult Epidermis. PLoS One 3, e1483. 10.1371/journal.pone.0001483 18213391 PMC2198947

[B102] Papayannopoulos V. (2018). Neutrophil Extracellular Traps in Immunity and Disease. Nat. Rev. Immunol. 18, 134–147. 10.1038/nri.2017.105 28990587

[B103] Parker H. Dragunow M. Hampton M. B. Kettle A. J. Winterbourn C. C. (2012). Requirements for NADPH Oxidase and Myeloperoxidase in Neutrophil Extracellular Trap Formation Differ Depending on the Stimulus. J. Leukoc. Biol. 92, 841–849. 10.1189/jlb.1211601 22802447

[B104] Paus R. Cotsarelis G. (1999). The Biology of Hair Follicles. N. Engl. J. Med. 341, 491–497. 10.1056/nejm199908123410706 10441606

[B105] Paus R. Nickoloff B. Ito T. (2005). A ?hairy? Privilege. Trends Immunol. 26, 32–40. 10.1016/j.it.2004.09.014 15629407

[B106] Paus R. van der Veen C. Eichmüller S. Kopp T. Hagen E. Müller-Röver S. (1998). Generation and Cyclic Remodeling of the Hair Follicle Immune System in Mice. J. Invest. Dermatol. 111, 7–18. 10.1046/j.1523-1747.1998.00243.x 9665380

[B107] Petukhova L. Duvic M. Hordinsky M. Norris D. Price V. Shimomura Y. (2010). Genome-wide Association Study in Alopecia Areata Implicates Both Innate and Adaptive Immunity. Nature 466, 113–117. 10.1038/nature09114 20596022 PMC2921172

[B108] Pinegin B. Vorobjeva N. Pinegin V. (2015). Neutrophil Extracellular Traps and Their Role in the Development of Chronic Inflammation and Autoimmunity. Autoimmun. Rev. 14, 633–640. 10.1016/j.autrev.2015.03.002 25797532

[B109] Pozdnyakova O. Mahalingam M. (2008). Involvement of the Bulge Region in Primary Scarring Alopecia. J. Cutan. Pathol. 35, 922–925. 10.1111/j.1600-0560.2007.00937.x 18537862

[B110] Proost P. Loos T. Mortier A. Schutyser E. Gouwy M. Noppen S. (2008). Citrullination of CXCL8 by Peptidylarginine Deiminase Alters Receptor Usage, Prevents Proteolysis, and Dampens Tissue Inflammation. J. Exp. Med. 205, 2085–2097. 10.1084/jem.20080305 18710930 PMC2526203

[B111] Raijmakers R. Zendman A. J. W. Egberts W. V. Vossenaar E. R. Raats J. Soede-Huijbregts C. (2007). Methylation of Arginine Residues Interferes with Citrullination by Peptidylarginine Deiminases *In Vitro* . J. Mol. Biol. 367, 1118–1129. 10.1016/j.jmb.2007.01.054 17303166

[B112] Reithmayer K. Meyer K. C. Kleditzsch P. Tiede S. Uppalapati S. K. Gläser R. (2009). Human Hair Follicle Epithelium Has an Antimicrobial Defence System that Includes the Inducible Antimicrobial Peptide Psoriasin (S100A7) and RNase 7. Br. J. Dermatol. 161, 78–89. 10.1111/j.1365-2133.2009.09154.x 19416233

[B113] Rinaldi L. Avgustinova A. Martín M. Datta D. Solanas G. Prats N. (2017). Loss of Dnmt3a and Dnmt3b Does Not Affect Epidermal Homeostasis but Promotes Squamous Transformation through PPAR-γ. Elife 6, e21697. 10.7554/eLife.21697 28425913 PMC5429093

[B114] Rinaldi L. Datta D. Serrat J. Morey L. Solanas G. Avgustinova A. (2016). Dnmt3a and Dnmt3b Associate with Enhancers to Regulate Human Epidermal Stem Cell Homeostasis. Cell Stem Cell 19, 491–501. 10.1016/j.stem.2016.06.020 27476967

[B115] Rogers G. E. Harding H. W. J. Llewellyn-Smith I. J. (1977). The Origin of Citrulline-Containing Proteins in the Hair Follicle and the Chemical Nature of Trichohyalin, an Intracellular Precursor. Biochim. Biophys. Acta (Bba) - Protein Struct. 495, 159–175. 10.1016/0005-2795(77)90250-1 410454

[B116] Rosazza T. Warner J. Sollberger G. (2021). NET Formation - Mechanisms and How They Relate to Other Cell Death Pathways. FEBS J. 288, 3334–3350. 10.1111/febs.15589 33047496

[B117] Rosenblum M. D. Olasz E. B. Yancey K. B. Woodliff J. E. Lazarova Z. Gerber K. A. (2004). Expression of CD200 on Epithelial Cells of the Murine Hair Follicle: a Role in Tissue-specific Immune Tolerance. J. Invest. Dermatol. 123, 880–887. 10.1111/j.0022-202x.2004.23461.x 15482475

[B118] Rosenblum M. D. Yancey K. B. Olasz E. B. Truitt R. L. (2006). CD200, a "no Danger" Signal for Hair Follicles. J. Dermatol. Sci. 41, 165–174. 10.1016/j.jdermsci.2005.11.003 16386879

[B119] Saijo S. Nagai A. Kinjo S. Mashimo R. Akimoto M. Kizawa K. (2016). Monomeric Form of Peptidylarginine Deiminase Type I Revealed by X-ray Crystallography and Small-Angle X-ray Scattering. J. Mol. Biol. 428, 3058–3073. 10.1016/j.jmb.2016.06.018 27393304

[B120] Schön M. P. Erpenbeck L. (2018). The Interleukin-23/Interleukin-17 Axis Links Adaptive and Innate Immunity in Psoriasis. Front. Immunol. 9, 1323. 10.3389/fimmu.2018.01323 29963046 PMC6013559

[B121] Shang Y. Hu X. DiRenzo J. Lazar M. A. Brown M. (2000). Cofactor Dynamics and Sufficiency in Estrogen Receptor-Regulated Transcription. Cell 103, 843–852. 10.1016/s0092-8674(00)00188-4 11136970

[B122] Shao S. Fang H. Dang E. Xue K. Zhang J. Li B. (2019). Neutrophil Extracellular Traps Promote Inflammatory Responses in Psoriasis via Activating Epidermal TLR4/IL-36R Crosstalk. Front. Immunol. 10, 746. 10.3389/fimmu.2019.00746 31024570 PMC6460719

[B123] Sharma P. Azebi S. England P. Christensen T. Møller-Larsen A. Petersen T. (2012). Citrullination of Histone H3 Interferes with HP1-Mediated Transcriptional Repression. Plos Genet. 8, e1002934. 10.1371/journal.pgen.1002934 23028349 PMC3441713

[B124] Slade D. J. Fang P. Dreyton C. J. Zhang Y. Fuhrmann J. Rempel D. (2015). Protein Arginine Deiminase 2 Binds Calcium in an Ordered Fashion: Implications for Inhibitor Design. ACS Chem. Biol. 10, 1043–1053. 10.1021/cb500933j 25621824 PMC4569063

[B125] Stadler S. C. Vincent C. T. Fedorov V. D. Patsialou A. Cherrington B. D. Wakshlag J. J. (2013). Dysregulation of PAD4-Mediated Citrullination of Nuclear GSK3 Activates TGF- Signaling and Induces Epithelial-To-Mesenchymal Transition in Breast Cancer Cells. Proc. Natl. Acad. Sci. 110, 11851–11856. 10.1073/pnas.1308362110 23818587 PMC3718105

[B126] Steinert P. M. Parry D. A. D. Marekov L. N. (2003). Trichohyalin Mechanically Strengthens the Hair Follicle. J. Biol. Chem. 278, 41409–41419. 10.1074/jbc.m302037200 12853460

[B127] Struyf S. Noppen S. Loos T. Mortier A. Gouwy M. Verbeke H. (2009). Citrullination of CXCL12 Differentially Reduces CXCR4 and CXCR7 Binding with Loss of Inflammatory and Anti-HIV-1 Activity via CXCR4. J. Immunol. 182, 666–674. 10.4049/jimmunol.182.1.666 19109200

[B128] Sun B. Chang H. H. Salinger A. Tomita B. Bawadekar M. Holmes C. L. (2019). Reciprocal Regulation of Th2 and Th17 Cells by PAD2-Mediated Citrullination. JCI insight 4, e129687. 10.1172/jci.insight.129687 PMC6948856 31723060

[B129] Sun B. Dwivedi N. Bechtel T. J. Paulsen J. L. Muth A. Bawadekar M. (2017). Citrullination of NF-Κb P65 Promotes its Nuclear Localization and TLR-Induced Expression of IL-1β and TNFα. Sci. Immunol. 2, eaal3062. 10.1126/sciimmunol.aal3062 28783661 PMC5718838

[B130] Tanemura A. Oiso N. Nakano M. Itoi S. Kawada A. Katayama I. (2013). Alopecia Areata: Infiltration of Th17 Cells in the Dermis, Particularly Around Hair Follicles. Dermatology 226, 333–336. 10.1159/000350933 23838575

[B131] Tanikawa C. Ueda K. Suzuki A. Iida A. Nakamura R. Atsuta N. (2018). Citrullination of RGG Motifs in FET Proteins by PAD4 Regulates Protein Aggregation and ALS Susceptibility. Cel Rep. 22, 1473–1483. 10.1016/j.celrep.2018.01.031 29425503

[B132] Tarcsa E. Marekov L. N. Andreoli J. Idler W. W. Candi E. Chung S.-I. (1997). The Fate of Trichohyalin. J. Biol. Chem. 272, 27893–27901. 10.1074/jbc.272.44.27893 9346937

[B133] Tarcsa E. Marekov L. N. Mei G. Melino G. Lee S.-C. Steinert P. M. (1996). Protein Unfolding by Peptidylarginine Deiminase. J. Biol. Chem. 271, 30709–30716. 10.1074/jbc.271.48.30709 8940048

[B134] Thiam H. R. Wong S. L. Qiu R. Kittisopikul M. Vahabikashi A. Goldman A. E. (2020). NETosis Proceeds by Cytoskeleton and Endomembrane Disassembly and PAD4-Mediated Chromatin Decondensation and Nuclear Envelope Rupture. Proc. Natl. Acad. Sci. USA 117, 7326–7337. 10.1073/pnas.1909546117 32170015 PMC7132277

[B135] Tillack K. Breiden P. Martin R. Sospedra M. (2012). T Lymphocyte Priming by Neutrophil Extracellular Traps Links Innate and Adaptive Immune Responses. J.I. 188, 3150–3159. 10.4049/jimmunol.1103414 22351936

[B136] Tilvawala R. Nguyen S. H. Maurais A. J. Nemmara V. V. Nagar M. Salinger A. J. (2018). The Rheumatoid Arthritis-Associated Citrullinome. Cel Chem. Biol. 25, 691–704. 10.1016/j.chembiol.2018.03.002 PMC6014894 29628436

[B137] Tsourouktsoglou T.-D. Warnatsch A. Ioannou M. Hoving D. Wang Q. Papayannopoulos V. (2020). Histones, DNA, and Citrullination Promote Neutrophil Extracellular Trap Inflammation by Regulating the Localization and Activation of TLR4. Cel Rep. 31, 107602. 10.1016/j.celrep.2020.107602 32375035

[B138] Tutturen A. E. V. Fleckenstein B. de Souza G. A. (2014). Assessing the Citrullinome in Rheumatoid Arthritis Synovial Fluid with and without Enrichment of Citrullinated Peptides. J. Proteome Res. 13, 2867–2873. 10.1021/pr500030x 24724574

[B139] Ü. Basmanav F. B. Cau L. Tafazzoli A. Méchin M.-C. Wolf S. Romano M. T. (2016). Mutations in Three Genes Encoding Proteins Involved in Hair Shaft Formation Cause Uncombable Hair Syndrome. Am. J. Hum. Genet. 99, 1292–1304. 10.1016/j.ajhg.2016.10.004 27866708 PMC5142115

[B140] Van Avondt K. Hartl D. (2018). Mechanisms and Disease Relevance of Neutrophil Extracellular Trap Formation. Eur. J. Clin. Invest. 48 (Suppl. 2), e12919. 10.1111/eci.12919 29543328

[B141] van Beers J. J. B. C. Schwarte C. M. Stammen-Vogelzangs J. Oosterink E. Božič B. Pruijn G. J. M. (2013). The Rheumatoid Arthritis Synovial Fluid Citrullinome Reveals Novel Citrullinated Epitopes in Apolipoprotein E, Myeloid Nuclear Differentiation Antigen, and β-actin. Arthritis Rheum. 65, 69–80. 10.1002/art.37720 23044660

[B142] Vossenaar E. R. Zendman A. J. W. van Venrooij W. J. Pruijn G. J. M. (2003). PAD, a Growing Family of Citrullinating Enzymes: Genes, Features and Involvement in Disease. Bioessays 25, 1106–1118. 10.1002/bies.10357 14579251

[B143] Wang Y. Wysocka J. Sayegh J. Lee Y. H. Perlin J. R. Leonelli L. (2004). Human PAD4 Regulates Histone Arginine Methylation Levels via Demethylimination. Science 306, 279–283. 10.1126/science.1101400 15345777

[B144] Wang Y. Li M. Stadler S. Correll S. Li P. Wang D. (2009). Histone Hypercitrullination Mediates Chromatin Decondensation and Neutrophil Extracellular Trap Formation. J. Cel Biol 184, 205–213. 10.1083/jcb.200806072 PMC2654299 19153223

[B145] Wang Y. Wysocka J. Sayegh J. Lee Y.-H. Perlin J. R. Leonelli L. (2004). Human PAD4 Regulates Histone Arginine Methylation Levels via Demethylimination. Science 306, 279–283. 10.1126/science.1101400 15345777

[B146] Winget J. M. Finlay D. Mills K. J. Huggins T. Bascom C. Isfort R. J. (2016). Quantitative Proteomic Analysis of Stratum Corneum Dysfunction in Adult Chronic Atopic Dermatitis. J. Invest. Dermatol. 136, 1732–1735. 10.1016/j.jid.2016.03.037 27091361 PMC5018406

[B147] Winter M. Moser M. A. Meunier D. Fischer C. Machat G. Mattes K. (2013). Divergent Roles of HDAC1 and HDAC2 in the Regulation of Epidermal Development and Tumorigenesis. EMBO J. 32, 3176–3191. 10.1038/emboj.2013.243 24240174 PMC3981143

[B148] Witalison E. Thompson P. Hofseth L. (2015). Protein Arginine Deiminases and Associated Citrullination: Physiological Functions and Diseases Associated with Dysregulation. Cdt 16, 700–710. 10.2174/1389450116666150202160954 PMC4520219 25642720

[B156] Wu J. Katrekar A. Honigberg L. A. Smith A. M. Conn M. T. Tang J. (2006). Identification of substrates of human protein-tyrosine phosphatase PTPN22. J. Biol. Chem. 281 (16), 11002–11010. 10.1074/jbc.M600498200 16461343

[B149] Yang M.-L. Sodré F. M. C. Mamula M. J. Overbergh L. (2021). Citrullination and PAD Enzyme Biology in Type 1 Diabetes - Regulators of Inflammation, Autoimmunity, and Pathology. Front. Immunol. 12, 678953. 10.3389/fimmu.2021.678953 34140951 PMC8204103

[B150] Ying S. Kojima T. Kawada A. Nachat R. Serre G. Simon M. (2010). An Intronic Enhancer Driven by NF-Κb Contributes to Transcriptional Regulation of Peptidylarginine Deiminase Type I Gene in Human Keratinocytes. J. Invest. Dermatol. 130, 2543–2552. 10.1038/jid.2010.179 20596086

[B151] Young C. Russell J. R. Lawson H. Mapperley C. Kranc K. R. Christophorou M. A. (2021). Peptidylarginine Deiminase IV (PADI4) Is Not Essential for Cell-Autonomous HSC Maintenance and normal Haematopoiesis. bioRxiv. 10.1101/2021.04.13.439513

[B152] Zeng Y. Ren R. Kaur G. Hardikar S. Ying Z. Babcock L. (2020). The Inactive Dnmt3b3 Isoform Preferentially Enhances Dnmt3b-Mediated DNA Methylation. Genes Dev. 34, 1546–1558. 10.1101/gad.341925.120 33004415 PMC7608744

[B153] Zhang X. Gamble M. J. Stadler S. Cherrington B. D. Causey C. P. Thompson P. R. (2011). Genome-wide Analysis Reveals PADI4 Cooperates with Elk-1 to Activate C-Fos Expression in Breast Cancer Cells. Plos Genet. 7, e1002112. 10.1371/journal.pgen.1002112 21655091 PMC3107201

[B154] Zhang X. Liu X. Zhang M. Li T. Muth A. Thompson P. R. (2016). Peptidylarginine Deiminase 1-catalyzed Histone Citrullination Is Essential for Early Embryo Development. Sci. Rep. 6, 38727. 10.1038/srep38727 27929094 PMC5144008

[B155] Zhou Y. Chen B. Mittereder N. Chaerkady R. Strain M. An L.-L. (2017). Spontaneous Secretion of the Citrullination Enzyme PAD2 and Cell Surface Exposure of PAD4 by Neutrophils. Front. Immunol. 8, 1200. 10.3389/fimmu.2017.01200 28993780 PMC5622307

